# Photogrammetry of Ultrafast Excited-State Intramolecular Proton Transfer Pathways in the Fungal Pigment Draconin Red

**DOI:** 10.3390/molecules28083506

**Published:** 2023-04-16

**Authors:** Janak Solaris, Taylor D. Krueger, Cheng Chen, Chong Fang

**Affiliations:** Department of Chemistry, Oregon State University, 153 Gilbert Hall, Corvallis, OR 97331, USA; dunnwalj@oregonstate.edu (J.S.); kruegeta@oregonstate.edu (T.D.K.); chenc9@oregonstate.edu (C.C.)

**Keywords:** dyes and pigments, naphthoquinones, ultrafast spectroscopy, quantum chemical calculations, bidirectional proton transfer, photochemistry, femtosecond stimulated Raman, organic materials

## Abstract

Proton transfer processes of organic molecules are key to charge transport and photoprotection in biological systems. Among them, excited-state intramolecular proton transfer (ESIPT) reactions are characterized by quick and efficient charge transfer within a molecule, resulting in ultrafast proton motions. The ESIPT-facilitated interconversion between two tautomers (PS and PA) comprising the tree fungal pigment Draconin Red in solution was investigated using a combination of targeted femtosecond transient absorption (fs-TA) and excited-state femtosecond stimulated Raman spectroscopy (ES-FSRS) measurements. Transient intensity (population and polarizability) and frequency (structural and cooling) dynamics of –COH rocking and –C=C, –C=O stretching modes following directed stimulation of each tautomer elucidate the excitation-dependent relaxation pathways, particularly the bidirectional ESIPT progression out of the Franck–Condon region to the lower-lying excited state, of the intrinsically heterogeneous chromophore in dichloromethane solvent. A characteristic overall excited-state PS-to-PA transition on the picosecond timescale leads to a unique “W”-shaped excited-state Raman intensity pattern due to dynamic resonance enhancement with the Raman pump–probe pulse pair. The ability to utilize quantum mechanics calculations in conjunction with steady-state electronic absorption and emission spectra to induce disparate excited-state populations in an inhomogeneous mixture of similar tautomers has broad implications for the modeling of potential energy surfaces and delineation of reaction mechanisms in naturally occurring chromophores. Such fundamental insights afforded by in-depth analysis of ultrafast spectroscopic datasets are also beneficial for future development of sustainable materials and optoelectronics.

## 1. Introduction

Organic dyes with excited-state intramolecular proton transfer (ESIPT) capabilities have long been in the spotlight of energy transfer research due to their high degree of photostability, efficiency of charge transfer, low costs relative to inorganic materials, production and waste sustainability [1,2,3,4,5,6,7,8,9,10]. This unique class of pigments contains many naturally occurring compounds that may be extracted from plants or fungi such as xylindein (a blue-green pigment secreted by fungi in the *Chlorociboria* genus) [11,12,13,14,15,16] and alizarin (a red pigment from the root of the madder plant) [17,18,19]. ESIPT dyes exhibit several common traits indicative of their characteristic isomerization, including large Stokes shifts and tautomer-overlapping steady-state emission profiles that result from the complex excited-state potential energy landscapes, which have been extensively studied using experimental and computational approaches [9,20,21,22,23,24,25,26,27,28,29,30,31,32,33]. In the solid state, ESIPT dyes have been utilized to make a wide variety of optoelectronics including solar cells and organic light-emitting diodes (OLEDs) contained in consumer electronics, as well as organic semiconductors [1,3,14,34,35,36].

Among the ESIPT dyes, Draconin Red is a pigment extracted from the fungus *Scytalidium cuboideum* [37,38,39], which is notable in its molecular structure wherein ESIPT can occur at two atomic sites [38,40,41,42], reminiscent of the widely used dye indigo (see a salient comparison between indigo/alizarin and Draconin Red in Section 3 below) [43,44,45]. However, while both indigo and Draconin Red have two ESIPT sites and a high degree of symmetry, Draconin Red’s ketonic nature means that two separate proton transfer events (one at each ESIPT site) will result in a reformation of the original (pre-ESIPT) structure (see below).

**Scheme 1:**
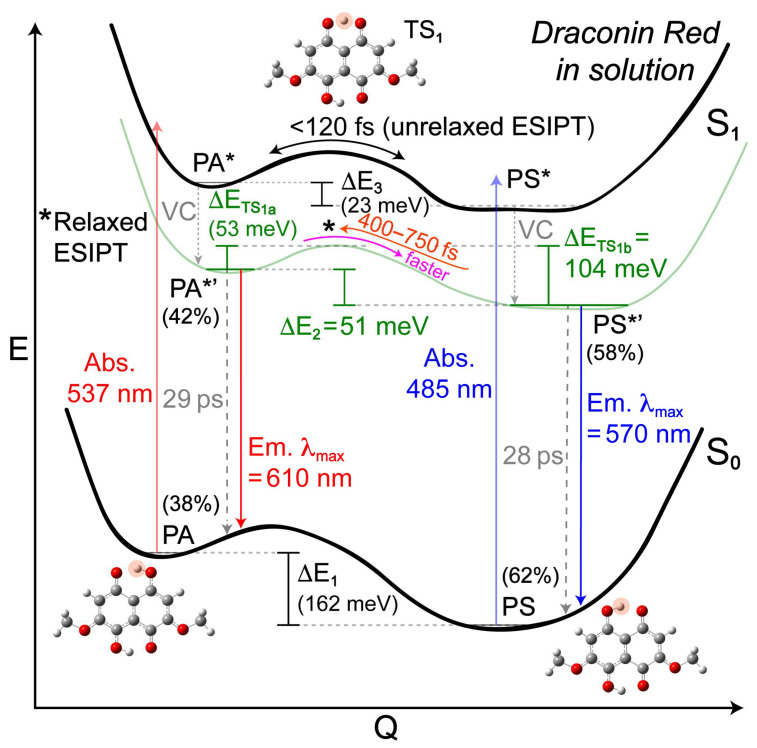
Ground- and excited-state potential energy surfaces of Draconin Red in solution. See Figure 1a,b for the two interconverting tautomers and Figure 1c for their absorption and emission profiles. The calculated energy differences (in milli-electron volt unit) between various tautomer states are denoted, with TS_1a_ and TS_1b_ representing two transition pathways (i.e., the associated ΔE values are listed in green color for the relaxed excited states of both tautomers). The predicted tautomer population percentages are listed in parentheses by the ground states (PA and PS) and relaxed excited states (PA*’ and PS*’). Solid upward and downward arrows depict electronic absorption (485 nm, violet; 537 nm, orange) and emission (570 nm, blue; 610 nm, red), respectively, while gray dotted and dashed downward arrows show vibrational cooling (VC) and nonradiative relaxation pathways. The unrelaxed and relaxed ESIPT pathways are labeled, with key time constants listed. Adapted with permission from [46]. Copyright 2021 American Chemical Society.

Our previous work on Draconin Red unveiled that the uniquely shaped steady-state electronic spectral profiles (both absorption and emission) result from a combination of an inherently heterogeneous population of tautomers as well as features attributed to vibronic coupling effects [46]. The more stable tautomeric form of Draconin Red is dubbed *para*-symmetric (PS) according to the ketone groups placed on two opposite carbon atoms in one aromatic ring. The other tautomer, which comprises an estimated 38% of the ground-state population, is the *para*-antisymmetric or PA tautomer, with ketone groups located on two carbon atoms at opposite ends of two aromatic rings (essentially in a centrosymmetric geometry except the two methoxy sidechains). We performed quantum calculations and predicted an energy barrier for keto-enol tautomerization involving the transfer of either hydroxy proton to its adjacent ketonic oxygen; for the relaxed excited state of PA*’ tautomer (the asterisk denotes an excited state, and the prime denotes a relaxed state), the transfer of a proton proximal to the methoxy groups requires ~137 meV of activation energy, whereas the proton distal to the methoxy groups requires ~104 meV (see Section 2.1 and Scheme 1 below). In the electronic ground state (S_0_), the energy barrier for PS to undergo proton transfer is raised by the difference in energy between the tautomers: 162 meV, meaning that the energy required to convert PS into PA in S_0_ is more than double the one required to convert PA into PS. Notably, such a transition barrier is lowered considerably in the excited state—directly following a vertical excitation of PS into a singlet excited state termed PS*, it is only 23 meV more stable than its counterpart, PA*. Once cooled out of this initially populated, vibrationally “hot” state into the more relaxed PS*’, the energy difference between the two tautomers grows to 51 meV. This stability difference is nearly equal to the activation energy of 53 meV to undergo downhill tautomerization from PA*’ to PS*’, which involves movement of a proton distal to the methoxy groups due to the aforementioned trend in activation energy. Moreover, there is an overall “bottleneck” for the more uphill tautomerization from PS*’ to PA*’ which allows us to track the molecular transformation (tautomerization here) with sufficient structural and temporal resolutions with a suitable spectroscopic toolset [47]. The higher degree of parity between the two tautomers in the S_1_ state (than S_0_ state) is reflected in the steady-state emission spectrum—summation of the vibronically resolved calculated spectra for two tautomers closely matches the measured overall emission spectrum with a PA*’:PS*’ population ratio of 42%:58%, contrasting with the ground-state PA:PS population ratio of 38%:62%, essentially meaning that some PS*/PS*’ must convert to PA*/PA*’ populations in the excited state [46]. We remark that these systematic calculations are mainly used as a qualitative trend check, instead of the exact quantitative values (which can be improved by advanced theories and calculations to model/simulate the coupled motions of electrons and atoms on intrinsic molecular timescales in realistic environments [31,48,49,50,51,52,53,54,55,56], beyond our ultrafast experimental focus here). This earlier foundational work also shows that initial relaxation of the excited species is dependent on the wavelength of light used to stimulate the electronic transition, with higher energy (i.e., bluer) light more able to provide the energy required for an uphill conversion from PS*/PS*’ to PA*/PA*’ species, which motivates this study of light-dependent relaxation pathways by means of a powerful tabletop optical toolset of ultrafast electronic and vibrational spectroscopies [47,57,58,59,60,61].

In particular, femtosecond transient absorption (fs-TA) spectroscopy establishes the excited-state potential energy surface (PES) with key time constants retrieved from detailed comparisons between the probe-dependent fits focusing on specific tautomers and global analysis. Besides steady-state electronic measurements and quantum calculations (see Experimental Methods in Section 4.4 below), we performed the excited-state femtosecond stimulated Raman spectroscopy (ES-FSRS) to provide crucial structural dynamics insights especially on the fs to picosecond (ps) timescales [58,62,63,64,65]. Recent works used tunable Raman pump pulses and/or combined FSRS experimental results with quantum chemical calculations to shed new light on a wide range of chemical (e.g., molecular motors and environmental pollutants), biological (e.g., heme proteins and fluorescent proteins), and materials (e.g., conjugated organic polymers and chromophores) systems [66,67,68,69,70,71,72,73,74,75]. The engaged readers for technical details can refer to representative literature [47,59,61,76,77] for the advantages and current limitations for the mixed time-frequency-domain FSRS methodology and its purely time-domain analogue [78,79,80,81]. Nevertheless, the elucidation of molecular structural dynamics for new knowledge and design principles has been demonstrated for these powerful stimulated Raman techniques, while their future development remains a driving force for broad communities with strong physical chemistry and chemical physics perspectives. For the tree fungal pigment Draconin Red [38,46] in this work, the intrinsic symmetry and resultant complexity of the bidirectional ESIPT reaction between the excited-state tautomers (PA*↔PS* in the “unrelaxed” ESIPT regime, and PA*’↔PS*’ in the “relaxed” ESIPT regime) allows us to dissect the transient electronic and vibrational signatures, both from physical chemistry and ultrafast spectroscopy perspectives, tracking an ultrafast conversion between two tautomers (e.g., PA*’→PS*’ on the 280–550 fs timescale and PS*’→PA*’ on the ~1 ps timescale) prior to the onset of fluorescence. This work thus paints a more complete portrait of ESIPT reaction in Draconin Red, not only for two tautomers with better population distinction (afforded by strategic excitations) but also with molecular structural specificity (provided by femtosecond Raman), which underlies its working mechanisms and engineering potentials for broader applications of these fungal pigments as naturally sourced, sustainable optoelectronic materials.

## 2. Results and Discussion

### 2.1. Steady-State Electronic Spectroscopy and Femtosecond Transient Absorption Spectroscopy

**Figure 1:**
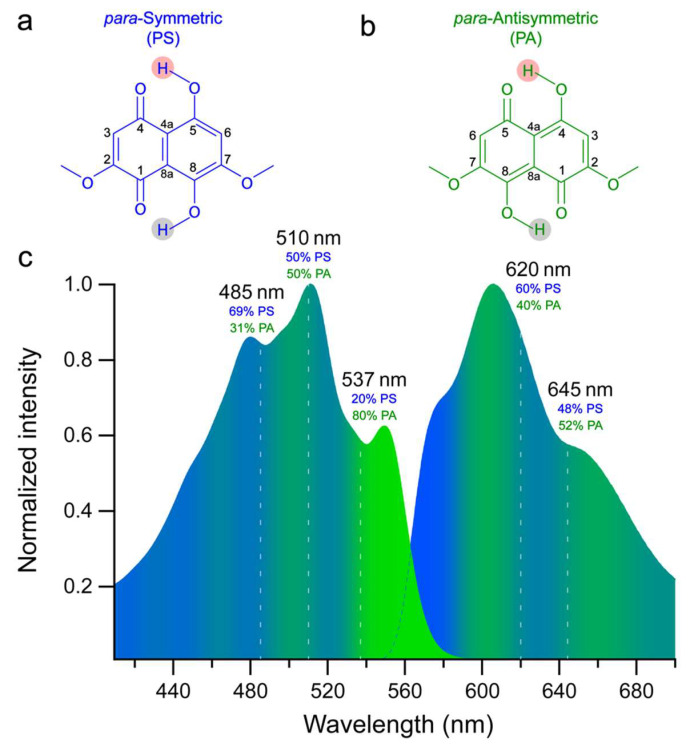
Structures of the two predominant tautomers of Draconin Red, dubbed *para*-symmetric or PS (**a**), and *para*-antisymmetric or PA (**b**). The protons that are proximal and distal to the methoxy sidechains are highlighted by semi-transparent gray and red circled shades, respectively. (**c**) The experimental steady-state electronic absorption and emission spectra of Draconin Red in DCM solvent are filled by colored shades at each wavelength according to an interpolated color scale of the calculated contribution from each tautomer, PS in blue and PA in green, with fine spectral features attributed to the vibronic bands. Vertical dashed lines indicate the experimentally relevant wavelengths for laser pulses used in fs-TA and FSRS measurements in this work, and the associated tautomer population percentages are listed nearby.

Since steady-state electronic absorption and emission spectra of Draconin Red in dichloromethane (DCM solvent) exhibit clear vibronic features from two closely related tautomers (Figure 1a,b), we performed suitable levels of quantum calculations [17,82,83,84,85,86,87,88,89] to estimate the relative populations at certain wavelengths selected in this work (Figure 1c). In the PS tautomer (Figure 1a), the ketone groups are placed on the first and fourth carbons on one aromatic ring so the two hydroxyl groups point their H atoms in the same direction. In the PA tautomer (Figure 1b), the ketone groups are placed on the first and fifth carbons on two aromatic rings so the two hydroxyl groups point their H atoms in the opposite directions. To perform time-resolved measurements on Draconin Red in solution, two actinic pump conditions were chosen for femtosecond transient absorption (fs-TA) spectroscopy. A 485 nm pump was used to target the S_0_→S_1_ electronic transition of the PS tautomer, while a 537 nm pump was used to target the corresponding transition in the PA tautomer. Due to their nearly identical chemical structures, PS and PA have similar electronic transition energies; considering the broad bandwidth of our fs actinic pump pulse in the optical setup (see Section 4.3 below), it is infeasible to excite one population completely independent of the other due to similar electronic transition energies of PS and PA with nearly identical chemical structures. Actinic pump conditions were strategically chosen with the intent of exciting the most unbalanced ratio of tautomers possible (e.g., ~70% PS at 485 nm versus 80% PA at 537 nm considering the peak excitation) at room temperature. In particular, the lower transition energy of PA lends itself well to this disparity: a 537 nm pump is predicted to excite a 4:1 ratio of PA:PS molecules. This strategy proved more difficult in exciting purely PS, as a bluer actinic pump risks exciting both tautomers to higher-lying electronic states. Therefore, the 485 nm actinic pump is predicted to excite a roughly 2:1 ratio of PS to PA, potentially leading to more spectral overlap issues and averaged data trends.

**Figure 2:**
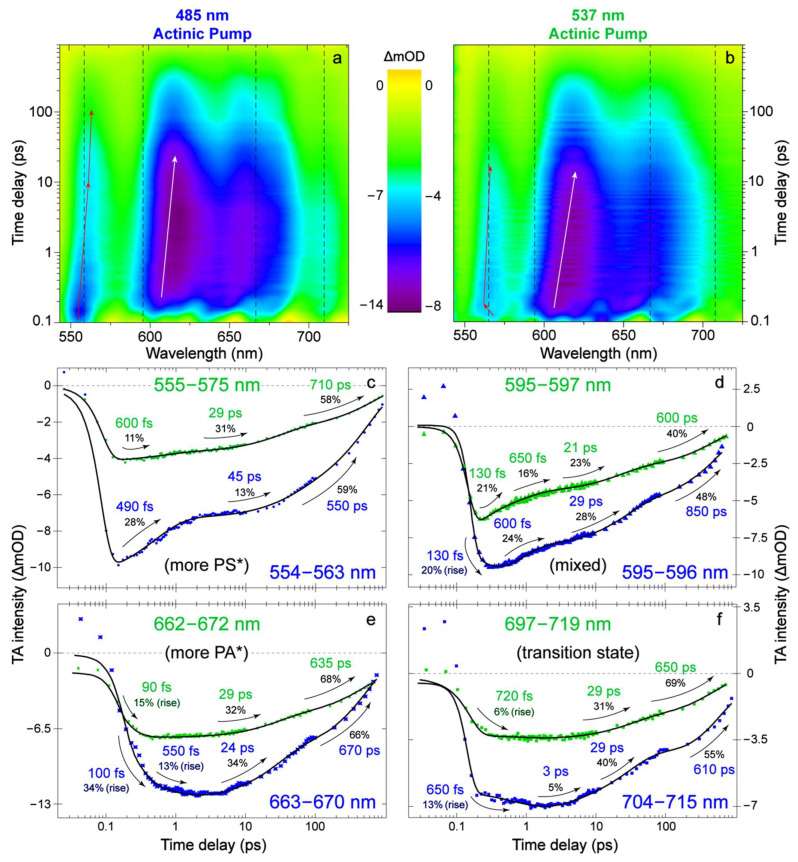
Transient electronic dynamics of Draconin Red in DCM solvent under two excitation conditions. Contour plots of fs-TA spectra following (**a**) 485 nm and (**b**) 537 nm excitation. Notable spectral regions of the stimulated emission (SE) bands were chosen for the probe-dependent fitting, marked by vertical dashed lines. Characteristic SE peak wavelength shifts are denoted by the titled thin red and white arrows for the minor and major SE bands, respectively. Spectral data (color-coded dots, triangles, and squares) integrated over certain regions (denoted in each panel) are overlaid with the least-squares fits (black solid curves) for the (**c**) mostly PS*-attributed blue SE band, (**d**) mixed SE band with a peak wavelength redshift, (**e**) red SE shoulder that is predicted to be mostly PA*, and (**f**) the farthest red SE band that is more pronounced after 485 nm excitation. Blue and green colors represent the 485 and 537 nm actinic pump conditions, respectively. The retrieved decay time constants and amplitude weights (summed to 100%) are listed by the best fits, with the signal intensity magnitude rise components denoted separately with their amplitude weights scaled to the summation of all the decay components. The zero-difference optical density (OD) is shown as a gray dashed line, and the TA signal intensity has the difference milli-OD (ΔmOD) unit.

**Figure 3:**
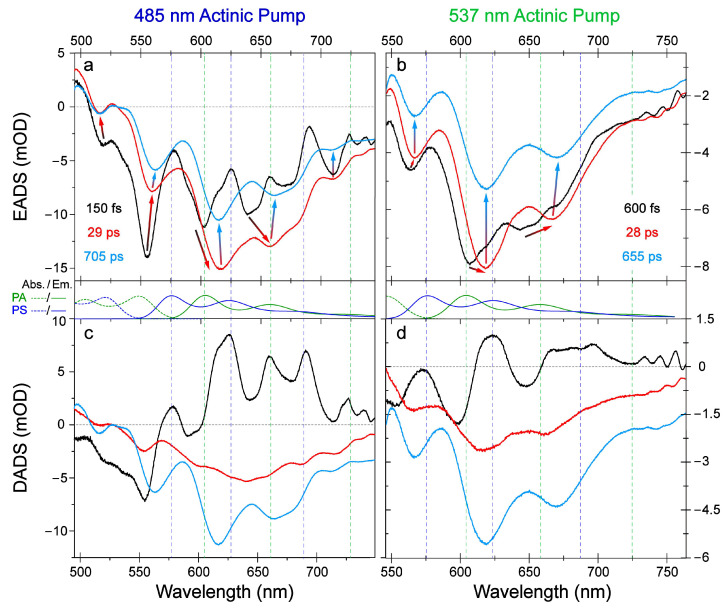
Global analysis of the fs-TA spectra of Draconin Red in DCM solvent following two representative excitation wavelengths. The evolution-associated difference spectra (EADS) after (**a**) 485 nm and (**b**) 537 nm excitations expose notable differences on the basis of a kinetic scheme: black→red→cyan traces, with the associated lifetimes listed in the insets. The color-coded arrows depict the spectral peak evolution across the detection window. The decay-associated difference spectra (DADS) after (**c**) 485 nm and (**d**) 537 nm excitations provide further insights on the basis of a parallel model with identical lifetimes as EADS. Middle panels show the calculated absorption (dotted lines) and emission (solid lines) bands of the PA (green) and PS (blue) tautomers. Vertical dashed lines are color-coded to highlight the corresponding PS* and PA* emission peak locations across the visible spectral window. The zero-difference in milli-optical density (mOD) is denoted by the horizontal dotted line in each panel (except in panel **b** where all signals are negative).

The TA spectrum of Draconin Red in DCM solvent is dominated by stimulated emission (SE) bands across the visible spectral region (Figure 2a,b), which are largely conserved with different actinic pump wavelengths on the fs to nanosecond (ns) timescales. A close inspection reveals different patterns of electronic dynamics following photoexcitation of the chromophore. Notably between the two excitation conditions used, a bluer actinic pump (485 nm, Figure 2a) causes an earlier onset and more pronounced redshift of the minor SE band at ~560 nm, but a smaller redshift of the major SE peak at ~610 nm (see the white arrow in Figure 2a) and its redder shoulder. This condition also generates a larger magnitude of signal difference at these SE peak locations (likely contributed by the higher absorbance at 485 nm than 537 nm, Figure 1c), as well as a stronger SE feature around 710 nm (see the weak cyan stripe in Figure 2a) which can be better viewed in the global analysis results (Figure 3a). The placement of the blue shoulder around 560 nm is similar to that in the steady-state emission spectrum, which is the region predicted to be the least mixed emission signal that arises almost purely from the PS tautomer (see Figure 1c). While this feature could have contributions from the ground-state bleaching of PA’s reddest absorption band, the clear dependence of this feature’s intensity and dynamics on the excitation wavelength (Figure 2a,b) indicates that it is predominantly an SE band from PS* species.

In particular, after 485 nm excitation, the SE peak rises at ~555 nm within 100 fs, then red-shifts to 561 nm around 10 ps, and finally to 563 nm around 100 ps. This pattern is highlighted by the black-to-red-to-cyan arrows in Figure 3a after peak deconvolution via global analysis. For comparison, after 537 nm excitation, a more stagnant SE peak emerges at ~568 nm, quickly blue-shifts to 562 nm within ~100 fs of reaching its maximum intensity magnitude, then slowly red-shifts to ~565 nm at 10 and 100 ps time delay points (also highlighted by arrows in Figure 3b). We note the ~100 fs timescale is approaching the pump–probe cross-correlation time of <140 fs (see experimental methods in Section 4.3 below), while the detailed fitting results for the probe-dependent TA data can be seen in Figure 2c–f. Taken together, these results indicate that the PS* population formed upon 485 nm excitation can efficiently relax into a lower-lying energy state termed PS*’ (Scheme 1). In other words, some initially excited PS* subpopulation does not tautomerize into PA*, but instead directly cools into PS*’ and leads to a continuous redshift with a significant intensity decay of the SE peak in the PS* emission range (see thin red arrows in Figure 2a, and color-coded arrows in Figure 3a). This interpretation is corroborated by an emergent redder and weaker SE peak at ~570 nm (closer to the PA* emission range) upon 537 nm excitation, which quickly blue-shifts to ~562 nm (before 150 fs, see the short red arrow in Figure 2b) which is closer to the PS* emission range, in accord with an ultrafast ESIPT event. When compared to the primary SE band in their respective experiments (Figure 2a,b), this minor SE band appears slightly later following 537 nm excitation, indicating that instead of tracking the cooling of PS* into PS*’ (Figure 2a) this peak follows a newly formed population of PS* from PA*, which then demonstrates a smaller magnitude of redshift as it further relaxes into PS*’ (see the long red arrow in Figure 2b, and color-coded arrows in Figure 3b). The end point for this specific SE peak redshift is similar (at ~565 nm at 100 ps) under both excitation conditions, supporting their common assignment to PS*’ (either after ultrafast ESIPT or semi-trapped in the corresponding excited state) that continues to relax into the ground state [30,61]. After 537 nm excitation, the adjacent main SE band above 600 nm displays a more prominent redshift on the corresponding timescale, as highlighted by the more tilted white arrow in Figure 2b than its counterpart in the same spectral region of Figure 2a. Furthermore, the disparity in magnitudes of the far-red SE peaks indicates a stronger signal from intermediate species after populating PS* states via 485 nm excitation (see Figure 2a and Figure 3a).

The least-squares multiexponential fits of several characteristic probe regions in the TA spectra provide useful insights into the relaxation mechanisms of Draconin Red after electronic excitation. The blue spectral region around 560 nm is predicted by our vibronic calculations to be mostly PS* emissions (Figure 1c and Figure 2c), the region around 595 nm that is on the blue side of the most intense SE peak (above 600 nm) contains more SE contribution from PA* (Figure 1c and Figure 2d), the red SE shoulder at 665 nm is associated with the second vibronic emission maximum of PA* (Figure 1c and Figure 2e), and the less intense redder SE shoulder at ~710 nm is likely attributable to an intermediate transition state (Figure 2f). Upon electronic excitation, regardless of the actinic pump wavelength used, both the probe-dependent fits (Figure 2c–f) and global analysis results (Figure 3) display characteristic electronic dynamics and universally show an apparent fluorescence lifetime on the order of 500–850 ps. Similarly, a rotational relaxation time of the chromophore ranging from ~21 to 45 ps is observed in each case, consistent with previously published results for Draconin Red in DCM solvent [46]. The major differences in TA spectra under the two excitation conditions can be found primarily on the sub-ps timescale with contrasting rise and decay patterns (e.g., time constants and amplitude weights, intensity magnitudes, and time delay in reaching the maximal intensity), which unveil the key to cartographic insights into the excited-state landscape of Draconin Red.

First, for the blue SE band around 560 nm with a predicted dominance from PS*, the excitation-dependent signal divergence before ~10 ps is apparent (Figure 2c). Importantly, we note a clear and consistent trend as the excitation wavelength is tuned: the ~560 nm SE peak intensity is stronger, similar in magnitude, and weaker than the main ~605 nm SE peak after 485, 510, and 537 nm excitation of Draconin Red in DCM solvent [46], confirming that a bluer pump across this ~50 nm range can yield more PS* species. In addition, the magnitude of the blue/red SE peak intensity decay/rise is the largest following 485 nm excitation and the smallest after 537 nm excitation (with 510 nm excitation in the middle), strongly suggesting an interconversion between these two electronic states which are responsible for the observed SE peak evolution pattern. The PA-targeted redder pump at 537 nm results in a longer and less pronounced (11%) decay component than the PS-targeted bluer pump at 485 nm (28% weight), while the signal intensity magnitude is consistently larger in the latter case for direct excitation, further supporting the PS* SE band assignment in this rather “clean” spectral region. The clear contrast in duration and magnitude of post-excitation signal decays indicate that the selected pump wavelengths can achieve detectable population difference of two closely related tautomers of Draconin Red from the equilibrium (ground-state thermal fluctuations) to non-equilibrium (undergoing ESIPT reaction) regimes. From the tautomer population partition in absorption profile (Figure 1c), 537 nm light primarily excites PA to PA*, and a small population of PS species can still be excited to PS*; however, 485 nm light swaps the primary tautomer excited, favoring PS* more than PA*. Due to challenges of predicting the exact transition oscillator strengths, we need to rely on the comparative TA data analysis across the detection window to assign major tautomer species. Besides the intensity dynamics (Figure 2c), the SE peak location dynamics on the fs-to-ps timescale provide further insights: 485 nm excitation directly generates PS* population closer to the photoexcitation time zero (Figure 2a and Figure 3a, see the prompt onset of the ~555 nm SE peak), whereas 537 nm excitation leads to a PS* population being generated primarily by way of PA*→PS* tautomerization (Figure 2b, see the left-pointing thin red arrow). This ultrafast interconversion step following 537 nm excitation also serves to explain the longer decay time constant (600 fs, due to the extra step of PA*→PS* transition; also see the retrieved lifetime in Figure 3b) than the counterpart following 485 nm excitation (490 fs, a more direct population of PS*; also see a shorter lifetime for the initial overall process in Figure 3a). The numerical difference between these two decay time constants is in accord with the expected <120 fs duration of a “hot” downhill ESIPT pathway between the unrelaxed PS* and PA* species immediately following photoexcitation, which is also consistent with previous experimental and computational studies of nonadiabatic ESIPT timescale of ~90 fs for 3-hydroxychromone that likely involves a photoexcited higher-lying S_2_ state [31,90] as well as ~97 fs ESIPT from the S_1_ state of normal form (9,10-quinone) to the tautomer form (1,10-quinone) of 1,8-dihydroxyanthraquinone (also called Dantron or chrysazin) in ethanol [30]. Note that in the latter case, ESIPT can only occur on one side of the chromophore due to the adjacent one carbonyl and two hydroxyl groups only on that side, in contrast to Draconin Red with two sides for ESIPT (though one side is slightly favored energetically according to our prior time-dependent density functional theory or TD-DFT calculations, see Scheme 1) [46].

Second, the specific probe window under investigation is on the blue side of the dominant SE peak at the emission maximum (~605 nm) predicted for PA*, so the PA* species can contribute to the dynamics observed, although this is a region with mixed contributions from both tautomers with broad and overlapping SE bands [46,61]. A smaller probe window with 1–2 nm width was used to better observe the tautomer-specific dynamics (Figure 2d). Notably, at this ~596 nm location, a 130 fs rise (20% weight) and decay (21% weight) component was retrieved after 485 and 537 nm excitation, respectively, indicative of an ultrafast ESIPT reaction on the 130 fs timescale that can be attributed to the PA*↔PS* transition after electronic excitation. Since the 485 nm light creates a population of PS* that tautomerizes into PA*, an initial rise in this emission range largely matches an initial decay following 537 nm excitation, thus reflecting the negligible energetic preference of tautomers in the initially accessed S_1_ state prior to vibrational cooling (see “VC” in Scheme 1) or other internal conversion on the sub-ps timescale. The subsequent decay time constant of ~600 fs is consistent with the previously reported ESIPT time constant in the relaxed S_1_′ state, referred to as an overall “bottleneck” time constant of 400–750 fs for the more uphill PS*’→PA*’ transition (Scheme 1). As the molecules vibrationally cool, the relative stability of the PS*’ configuration grows, contributing to the lengthened decay of SE band from both PA*’ and PS*’ beyond ~1 ps. Moreover, the amplitude weight of the 600 fs decay (24%) observed after 485 nm excitation is more than the corresponding weight of the 650 fs decay (16%) following 537 nm excitation, suggesting contributions from mixed tautomer species in this probe region where an SE peak redshift is visible (see Figure 2a,b and Figure 3a,b). Therefore, upon 485 nm excitation of the thermally equilibrated ~38%:62% PA:PS populations, some nascent PS* species undergo an uphill tautomerization into the marginally less stable PA* form, followed by vibrational cooling into PA*’ and a return to the ~42%:58% population ratio for PA*’:PS*’ (see steady-state emission spectrum in Figure 1c, and text above).

Third, in the PA*-emission-dominant probe window at ~665 nm, an ultrafast ~100 fs rise component can be retrieved under both excitation conditions in the “unrelaxed” ESIPT regime (Scheme 1). This rise component likely tracks the formation of unrelaxed PA* species from the initially populated PS*, which manifests a decay around this time in the ~560 nm probe window (see Figure 2c,e), again indicating that both PS* and PA* species are simultaneously excited. After 537 nm excitation, there is a PS* population (likely contributed by a prompt PA*→PS* transition within the instrument response function) that travels along the unrelaxed ESIPT pathway toward PA* on an ultrafast timescale before the subsequent decay processes, and the apparent ~90 fs rise time constant could be due to an overall population shift toward PA*’ as a red-emitting state (Scheme 1). Meanwhile, the direct excitation of PA* can also undergo ultrafast internal conversion to PA*’. This explanation is bolstered by the fact that the weight (34%) of the initial rise component after the bluer 485 nm excitation (i.e., generating more PS* to start with) is notably larger than its counterpart weight (15%) after the redder 537 nm excitation (Figure 2e). The 550 fs rise component seen in the former case likely tracks the PS*’-to-PA*’ tautomerization following vibrational cooling (i.e., the relaxed ESIPT pathway), since the bluer light generates much more PS* species that directly cools down to PS*’ which then undergoes the characteristic ~550 fs uphill transition toward PA*’. The similarity between the TA signal in this region after ~10 ps supports our assignment that a gradual redshift of the SE peak (highlighted by the black-to-red-to-cyan arrows around 650 nm in Figure 3a,b) corresponds to transient accumulation of more PA*’ species via the relaxed ESIPT following either bluer or redder excitations at 485 or 537 nm, as well as the subsequent energy dissipation pathways (denoted in Scheme 1) [46].

In the probe region above 700 nm, a clear SE peak can be seen after 485 nm excitation that is much less pronounced after 537 nm excitation (Figure 2a,b and Figure 3a,b). Both tautomers are calculated to have greatly diminished vibronic peaks in this region, so the far-red SE feature may arise from an intermediate state between ESIPT endpoints, with a proton shared between two semi-ketonic oxygens, as proposed in our previous work [46]. Although this TS_1_ state is higher in energy than either PA*’ or PS*’ states according to TD-DFT calculations in the relaxed excited state, it may be more easily accessed when the sample is exposed to higher-energy light, as is the case with 485 nm light to mainly excite PS species to PS* state. Interestingly, the 485 and 537 nm excitations result in small yet discernible rise components with 650 and 720 fs time constants, respectively, indicating that the underlying species is formed during an ESIPT process following most of the initial relaxation (e.g., vibrational-cooling-facilitated internal conversion from PS* to PS*’). This rise is larger in magnitude following 485 nm excitation since the more significant PS*’ population undergoes the uphill transition toward PA*’ via an intermediate state (e.g., see TS_1_ in Scheme 1) and consequently more of its signal can be measured. Furthermore, an extra ~3 ps decay component retrieved only after 485 nm excitation (Figure 2f) is similar to the solvation time of DCM (~2 ps), which may play a role in the relaxation of this intermediate species when more excess energy is available [91,92].

Considering the degree to which they conform to the predicted relaxation behavior of each excited-state population, the probe-dependent fits support the hypothesis that different actinic pumps can achieve targeted excitation of the two closely related tautomers of Draconin Red. Further evidence can be obtained via global analysis [93] of the TA spectra (Figure 3), resulting in the best-fit lifetimes that largely agree with the probe-dependent analysis (Figure 2). The kinetic three-component deconvolution under both 485 and 537 nm excitations yields an ESIPT-attributed component on the 150–600 fs timescale, a rotational relaxation component on the order of ~30 ps, and a sub-ns component that tracks the apparent fluorescence lifetime (i.e., due to all radiative and nonradiative pathways). The evolution-associated difference spectra (EADS, see black→red→cyan traces in Figure 3a,b) are sufficient in capturing the major relaxation pathways of Draconin Red undergoing ultrafast ESIPT prior to the rotational motion and other nonradiative relaxation (e.g., molecular collisions and heat dissipation) of Draconin Red in DCM solvent, besides its weak fluorescence (see Section 3 below). In particular, the characteristic SE peak shifts as denoted by color-coded arrows support the electronic state transition (internal conversion between various S_1_ states, Scheme 1) of two tautomers, aided by the quantum calculation results (see Figure 1c and middle panels in Figure 3). We note that adding more parameters to the fits typically led to fitting of the coherent artifact around time zero, and limiting these additional parameters would produce mirrored spectra, often less separated in time than the original data. Since the convoluted and heterogeneous nature of the analyte (i.e., two tautomers of Draconin Red) acts as a confounding factor [94,95], we focus on the least-squares fits with the minimal amount of parameters to gain essential electronic dynamics insights.

Interestingly, the decay-associated difference spectra (DADS), wherein positive and negative peaks in the same SE region respectively represent the increasing and decaying populations with a certain lifetime [65], bear clear resemblances to the emission spectra predicted by Franck–Condon vibronic analysis of each tautomer. After 485 nm excitation, the two pronounced positive peaks below 650 nm in the 150 fs spectrum (black trace, Figure 3c) correspond to the vibronic emission maxima of PS*’ tautomer (Scheme 1), indicating that a significant PS*’ population is being formed on this timescale due to ultrafast internal conversion from the effectively pumped PS*. There are also some positive peaks close to PA*’ emission maxima at ~605 and 660 nm, indicative of the unrelaxed ESIPT on this timescale as PS* and PA* populations concurrently undergo a single-step tautomerization after being excited by the higher-energy 485 nm light. The other noticeable positive feature around 725 nm may correlate to the aforementioned transition state TS_1_ during the unrelaxed ESIPT reaction (Scheme 1).

In contrast, after 537 nm excitation, the positive DADS with 600 fs lifetime more firmly points toward formation of a single tautomer, with a greatly diminished PA*’ signal around 665 nm. A minor PS*’ peak at ~625 nm is due to a much-reduced direct excitation of PS species by 537 nm light (Figure 1c). For the initial traces (black in Figure 3a,b), the significantly lengthened lifetime of 600 fs after 537 nm excitation (Figure 3d) versus 150 fs after 485 nm excitation (Figure 3c) reveals that a relaxed ESIPT was more retrievable from spectral data in the former case, wherein the redder light allows less PA*’ species to be generated on the more uphill transition timescale (see the orange curved arrow in Scheme 1) due to the significantly less PS*’ species generated. We note that an SE peak magnitude intensity rise is observed around 665 nm, albeit on a shorter timescale (90 fs) in the probe-dependent fit (Figure 2e), so the absence of a positive peak here in the 600 fs DADS (black trace, Figure 3d) implies some ultrafast PA*’ decay (essentially cancelling the early time rise component) besides spectral overlap with the adjacent negative peaks. This interpretation is in accord with the more downhill nature of the PA*/PA*’→PS*/PS*’ conversion, since a favorable reaction would proceed faster, rather than getting caught in the “bottleneck” of an uphill transition (e.g., see the curved orange and magenta arrows in Scheme 1). Moreover, the positive peak at 725 nm is absent from the black DADS (Figure 3d), or if present, much redder and less intense than its counterpart observed after 485 nm excitation (Figure 3c). This result substantiates the observation of an intermediate “transition state” during both the unrelaxed (Figure 3c) and relaxed (Figure 2f) ESIPT reaction mainly after the bluer-light irradiation.

Taken together, the probe-dependent fits and global analysis results of the excitation-wavelength-dependent fs-TA spectra delineate an initial interconversion between PS* and PA* tautomers (i.e., unrelaxed ESIPT) near the Franck–Condon region on the 100–150 fs timescale, a second observable interconversion route on the ~500–700 fs timescale (i.e., relaxed ESIPT) following ultrafast vibrational cooling to reach the PS*’ and PA*’ states, and the rotational relaxation and other long-time energy dissipation pathways on the ~29 ps and 550–850 ps timescales from the relaxed, weakly emissive PS*’ and PA*’ species of Draconin Red in solution. This systematic analysis of the photoinduced ESIPT pathways enriches Scheme 1 by providing additional electronic insights into the early time tautomerization with a closer correlation between the retrieved time constants and specific tautomers of the chromophore.

### 2.2. Femtosecond Stimulated Raman Spectroscopy (FSRS) of Draconin Red in Solution

**Figure 4:**
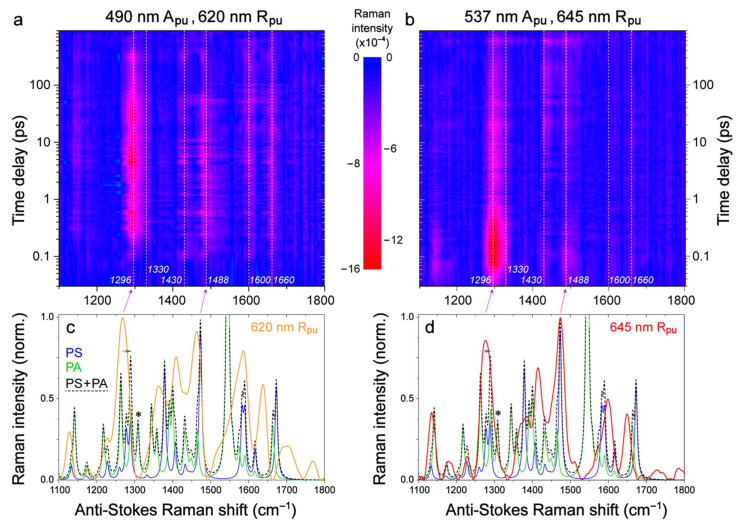
Femtosecond stimulated Raman spectroscopy (FSRS) of Draconin Red in DCM solvent from the electronic ground to excited states. The semilogarithmic 2D-contour plots of the excited-state (ES)-FSRS data (**a**) after a 490 nm actinic pump (A_pu_) paired with a 620 nm Raman pump (R_pu_), and (**b**) after 537 nm A_pu_ paired with a 645 nm R_pu_. The Raman probe is on the bluer side of R_pu_ to stimulate the Raman signal. The negative signal intensity color bar is shared in the middle. The prominent Raman peaks are highlighted by vertical white dashed lines with peak frequencies denoted. In lower panels, the corresponding ground-state (GS)-FSRS data are plotted in (**c**) and (**d**) with 620 nm R_pu_ (solid orange line) and 645 nm R_pu_ (solid red line), respectively, with the intensity multiplied by −1 for comparison to the calculated spectrum with positive intensity. The calculated ground-state Raman spectra for PS tautomer (blue solid), PA tautomer (green solid), and their sum (black dashed line) are overlaid with the GS-FSRS for a direct comparison. An asterisk denotes a calculated PA-specific mode at ~1310 cm^−1^, while the horizontal gradient gray arrow highlights a characteristic PA→PS peak frequency blueshift in a prominent marker-band region. The upward tilted magenta arrows between panels (**c**) and (**a**), as well as panels (**d**) and (**b**), highlight the photoinduced Raman peak frequency blueshift from the electronic ground state to excited state (close to the Franck–Condon region).

Though fs-TA spectroscopy is powerful in tracking the electronic dynamics of a photosensitive molecule like Draconin Red on intrinsic molecular timescales, transient vibrational spectroscopy can provide the useful structural dynamics insights with chemical bond precision (e.g., nuclear motions or normal modes) [47,59,96,97]. Guided by the locations of ground-state electronic bands particularly in the absorption regime (Figure 1c), we first performed the ground-state (GS)-FSRS measurements without a preceding actinic pump to capture vibrational marker bands at equilibrium (see Figure 4c,d). The 620 and 645 nm Raman pump wavelengths were selected on the red side of the broad absorption band to achieve largely absorptive lineshapes of the pre-resonantly enhanced FSRS signal, while the bluer probe on the anti-Stokes side (relevant discussions on terminology and applications in FSRS can be found in some reports [60,61,98,99,100]) covers a spectral range of ~558–580 and 578–602 nm (i.e., both in the PA-dominant absorption region, see Figure 1c) to collect the stimulated Raman signal from ~1100 to 1800 cm^−1^ (Figure 4c,d). In the excited-state (ES)-FSRS experiments following photoexcitation (i.e., an actinic pump or A_pu_), these two Raman pump (R_pu_) wavelengths can help to enhance the stimulated Raman signal from each tautomer species, on the basis of the positions of the emission peaks produced by vibronic analysis (Figure 1c). In particular, a 620 nm Raman pump is closer to resonance with the PS* tautomer, whereas a 645 nm Raman pump achieves more resonance with the PA* tautomer. The apparent difference between the ES-FSRS data collected under these two conditions (Figure 4a,b) substantiates the strategic tuning of incident laser wavelengths, mainly for the two pump pulses (i.e., A_pu_ and R_pu_), to enable the tautomer-specific tracking of structural dynamics undergoing ultrafast bidirectional ESIPT reaction for Draconin Red in solution. A general rule of thumb here is a bluer A_pu_ generates more PS* and PS*’ species, while a redder R_pu_ allows the dynamic resonance enhancement of more PA*’ species. Therefore, on the sub-ps timescale, a prominent peak rise and decay tracks the “unrelaxed” ESIPT reaction, whereas the “relaxed” ESIPT reaction dominates the apparent Raman peak intensity and frequency dynamics on the few ps to tens of ps timescales.

To help rationalize the measured spectra, density functional theory (DFT) calculations were performed to focus on the GS-FSRS data first (see Section 4.4 below). The Raman-active normal modes of each tautomer were calculated in the electronic ground state (S_0_). A scaling factor of 0.964 was multiplied to the frequencies (in cm^−1^ unit) of the predicted spectra to better match the observed Raman peak locations (Figure 4c,d). The most dominant feature at ~1275 cm^−1^ corresponds to similar vibrations in both tautomers, correlating to an asymmetric ring deformation in both PA and PS on the basis of ground-state calculations. In PA, the skeletal motion involves stretching of the C=C bonds at the 4th and 6th carbons (Figure 1b); in PS, the skeletal component is primarily ring deformation with C=C stretching near the two central carbons (labeled as 4a and 8a in Figure 1a) and C2–OMe stretch with notable C5–OH and C8–OH rocking motions (Figure 1a). Both tautomers see an asymmetric C–O–C stretch at one methoxy site coupled to a distal C–O–H rock, bringing the hydroxy proton on the 4th and 5th carbons in and out of proximity to the nearby ketone for PA and PS, respectively. In other words, this prominent vibration can serve as a marker band for proton motion [30,31,61], with the light-induced changes potentially reporting on the ESIPT process (see below) since the PA→PS transition can be generally correlated with the peak frequency blueshift (highlighted by the short horizontal arrow in Figure 4c). The bluer vibrational normal mode calculated for the PA tautomer at ~1310 cm^−1^ (see green solid trace with an asterisk in Figure 4c,d) is an in-plane asymmetric ring deformation mode that involves both hydroxy protons alternatingly displaced toward their own opposing ketone groups. The adjacent ~1345 cm^−1^ mode of PA has no direct analogue in PS, which may also contribute to the blue shoulder at ~1330 cm^−1^ in ES-FSRS (Figure 4a,b) but to a much less extent (vs. the lower-frequency PA modes) due to the calculated frequency separation and its weaker intensity compared to the 1263 cm^−1^ PA mode (see Figure 4c,d and more illustrations in the Supplementary Material).

On the higher-frequency side, two peaks in the excited state at ~1430 and 1488 cm^−1^ are likely related to the ground-state peaks at ~1400 and 1450 cm^−1^, with a frequency blueshift that is consistent with our prior report using a 537 nm actinic pump and a 645 nm Raman pump [46]. Both tautomers have the calculated vibrational normal modes around 1405 and 1436 cm^−1^, both of which may contribute to the observed excited-state peak around 1430 cm^−1^, but in each case of these analogous modes, PA is predicted to have a higher degree of polarizability and consequently a higher Raman signal intensity (Figure 4c,d). This imbalance informs the excited-state dynamics observed for this peak. Similarly, PS is predicted to have a Raman mode around 1470 cm^−1^ that is close to the 1488 cm^−1^ excited-state signal (denoted by a tilted magenta arrow between Figure 4a,c). PA has a comparatively weak Raman mode around 1450 cm^−1^ that may also contribute but to a much less extent. Meanwhile, the two tautomers’ vibrations in the 1400–1500 cm^−1^ spectral region contain many similarities, the foremost of which is an asymmetric stretch of conjugated bonds, causing skeletal deformation of the molecule. Generally speaking, the redder-frequency vibrations (those predicted to contribute to the 1430 cm^−1^ excited-state Raman feature) contain C–C–C asymmetric stretches of fractional first- to second-order bonds. For comparison, those vibrations in the bluer region predict the major displacement to be asymmetric stretches of similarly fractional C–C–OH bonds with a prominent COH rocking motion (see chemical structures of the two tautomers in Figure 1a,b).

Following 490 nm (Figure 4a) and 537 nm (Figure 4b) excitations, the ES-FSRS data are compared and contrasted with their corresponding GS-FSRS data in Figure 4c,d (see their spectral baselines in Figure S1b,c), respectively. The excited-state spectra in both cases display a noticeable frequency blueshift from the pronounced ground-state Raman peaks at ~1135, 1275, 1460, and 1635 cm^−1^, all of which involve COH rocking motions and hint at the prompt electronic redistribution about hydroxy groups when the chromophore is photoexcited, also in accord with the ultrafast ESIPT reaction. In particular, the high-frequency modes at ~1600 and 1660 cm^−1^ in ES-FSRS consist of significant C=O and ring C=C stretching motions which can be sensitive to the onset and ultrafast dynamics of ESIPT reaction progression (see Figures S1–S4 for the supporting ES-FSRS data analysis and discussions).

### 2.3. Revealing Bidirectional ESIPT during Excited-State Relaxation via Transient Raman Peak Dynamics

**Figure 5:**
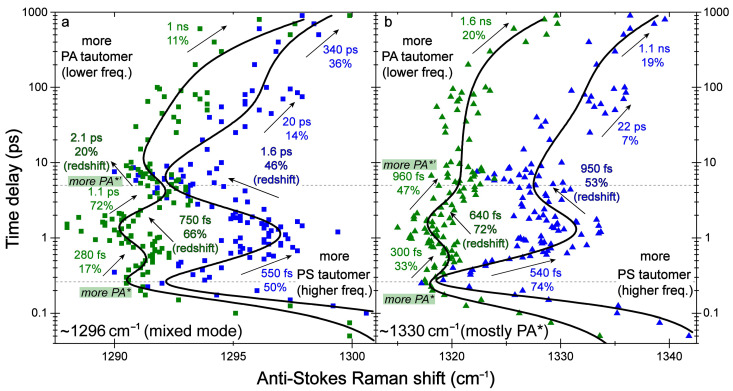
Structural dynamics traces obtained from ES-FSRS of Draconin Red in DCM solvent using a 490 nm actinic pump with a 620 nm Raman pump (blue) and a 537 nm actinic pump with a 645 nm Raman pump (green) for the (**a**) 1296 cm^−1^ (data points in squares) and (**b**) 1330 cm^−1^ (data points in triangles) modes. The pronounced 1296 cm^−1^ mode is comprised of contributions from a mixed tautomer population on the basis of quantum calculations, while the 1330 cm^−1^ shoulder peak is primarily associated with the PA* tautomer as the asterisk denotes an excited state species. The least-squares fits are shown in black lines to highlight the peak frequency dynamics, especially their similarities and differences between the two excitation-dependent experiments across the detection time window up to ~1 ns. The retrieved blueshift time constants and amplitude weights (summed to 100%) are listed by the best fits, with the peak frequency redshift components denoted separately with their amplitude weights scaled to the summation of all the blueshift components. The major turning points for the excited-state peak frequency dynamics are denoted by two horizontal dashed lines at ~250 fs and 5 ps with labels of more PA* and more PA*’, respectively.

**Figure 6:**
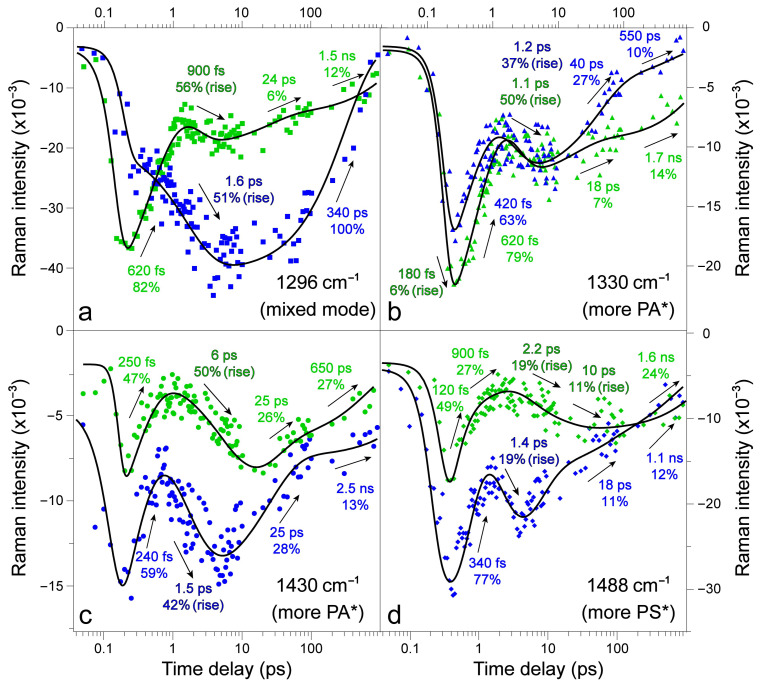
Transient Raman peak intensity dynamics from ES-FSRS of Draconin Red in DCM solvent obtained using a 490 nm actinic pump (A_pu_) paired with a 620 nm Raman pump (R_pu_) condition (blue) and a 537 nm A_pu_ paired with a 645 nm R_pu_ condition (green). The selective vibrational marker bands for analysis are marked by vertical dashed lines in Figure 4a,b for the (**a**) 1296 cm^−1^ mode that arises from a mixed tautomer population, (**b**) 1330 cm^−1^ and (**c**) 1430 cm^−1^ modes that arise from more PA* species in the excited state, and (**d**) 1488 cm^−1^ mode with more PS* contribution. The least-squares fits (black lines) are overlaid with scattered spectral data points in each panel. The retrieved intensity decay time constants and amplitude weights (summed to 100%) are listed by the best fits, with the signal intensity magnitude rise components denoted separately with their amplitude weights scaled to the summation of all the decay components. The stimulated Raman intensity axis is linear while the time delay axis is on the semilogarithmic scale.

Besides the two experimental conditions of 490 nm A_pu_, 620 nm R_pu_ (Figure 4a) and 537 nm A_pu_, 645 nm R_pu_ (Figure 4b) to target specific PS* and PA* tautomer species, we performed another experiment with a 485 nm A_pu_ and a 645 nm R_pu_ that results in a much lower signal intensity (Figure S2). This contrast showcases the importance of an optimal wavelength selection of A_pu_ and R_pu_ pairs to achieve the tracking specificity of transient molecular species. Notably, the ground-state spectrum was first subtracted from all the excited-state spectra, and broad spectral baselines were drawn to account for the R_pu_-affected TA profiles caused by the A_pu_. We also used semi-automatic baseline procedures with asymmetric linear-least-squares method in Origin software and TA spectra (see Section 2.1 above, and Figure S1a for representative spectra at selective time delay points) as guides in the manual baseline-drawing step in Igor software to eliminate parti pris influences, and the baseline-removed excited-state Raman peaks were then fit with Gaussian functions. The central location of each Gaussian peak as a function of time delay yields the excited-state mode frequency dynamics (see Figure 5 for representative cases), while the area/integrated intensity of each Gaussian peak as a function of time delay can be least-squares fitted with multiple exponentials to retrieve the lifetimes of contributing states in the non-equilibrium regime (Figure 6) [47,61]. The spectral regions of focus were selected on the basis of the observed signal magnitude and data spread in the region, so a more reliable peak analysis can be performed to yield robust structural dynamics insights. For example, the peak doublet around ~1600 and 1660 cm^−1^ lies on a sharp (sloped) region of the TA background after a 485–490 nm actinic pump thus making the peaks particularly prone to variance and difficult to analyze. We analyzed the ~1660 cm^−1^ mode intensity dynamics with a visibly large data spread (Figure S3) and focused on the major excitation-dependent trends while tracking the ESIPT reaction, for comparison with the other stronger Raman marker bands presented below (Figure 6). In general, ES-FSRS data require more components to fit satisfactorily than TA spectra, although they broadly agree with each other since the excited-state electronic dynamics hold for Draconin Red in solution, despite certain differences caused by the vibrational-mode-specific vibronic coupling and dynamic resonance conditions achieved during the ES-FSRS data collection.

Since the time-resolved excited-state Raman peak frequency directly reflects an intrinsic molecular property [47,59,101,102], instead of being affected by the electric polarizability and resonance conditions as the Raman peak intensity (see below) [60,103,104,105], we first examine the pronounced differences between the observed peak locations across the detection time window in the two excitation-dependent ES-FSRS measurements (Figure 5) to elucidate the structural dynamics of Draconin Red in solution. The key to understanding the processes underlying the peak frequencies is the interpretation of characteristic frequency shifts between ca. 200 fs and 10 ps when different pump conditions are used (see horizontal dashed lines in Figure 5). Upon photoexcitation, the excited-state spectrum bears a noticeable resemblance to the ground-state spectrum with the primary distinction being a nearly uniform blueshift of the entire spectrum (see Figure 4a,c and Figure 4b,d, also highlighted by two tilted magenta arrows for example). Most interestingly, the excited-state peaks emerge in these “blue-shifted” regions compared to GS-FSRS peaks, quickly red-shifting through early time delays till ~250 fs, followed by a blueshift in the range of a few hundred femtoseconds. We previously reported a ~20 cm^−1^ blueshift (1274→1294 cm^−1^) of the Raman marker band from ground state to excited state at 150 fs [46], which matches the aforementioned uniform blueshift of multiple Raman modes due to the light-induced electronic redistribution [61,102,106].

Notably, a systematic time-resolved study in this work resolved the initial peak frequency dynamics being a redshift in ES-FSRS as the photoexcited Draconin Red moves out of the Franck–Condon region, as shown by both the ~1296 and 1330 cm^−1^ modes in Figure 5 before ~250 fs. Since the quantum calculations consistently predict the PA peak frequency lower than the PS peak frequency in this region (Figure 4c,d and Figure S5), we can attribute the observed Raman band center frequency redshift to an ultrafast PS*→PA* transition within the instrument cross-correlation time (<140 fs) which is in the unrelaxed ESIPT regime (Scheme 1), and the subsequent frequency blueshift to an PA*’→PS*’ transition on the 280–550 fs timescale in the relaxed ESIPT regime along the more downhill pathway. In particular, there is an excitation-dependent signal divergence between ~250 fs and 5 ps for both modes analyzed: blueshift-redshift-blueshift with 537 nm A_pu_ and 645 nm R_pu_ vs. blueshift-redshift with 490 nm A_pu_ and 620 nm R_pu_ (see green vs. blue data points with best fits in black in Figure 5). The rapid frequency shifts observed using the 645 nm R_pu_ appear to correlate with those using the 620 nm R_pu_ but with additional features likely arising from the lower resonance specificity of the 645 nm R_pu_—hence multiple time constants retrieved from both “hot” tautomer contributions (Figure 1c) as well as an observed frequency trend being averaged that leads to reduced shift magnitudes. These analogous shifts indicate similar interconversions between the tautomers occurring in the range of 280–550 fs, while the faster “turnaround” with a 640–750 fs redshift component can be attributed to a more pronounced PA*’ formation via a more uphill ESIPT reaction from PS*’, in accord with our previously retrieved “bottleneck” time constant using fs-TA experiments with 510 and 537 nm A_pu_ [46]. In contrast, additional excitation energy provided by the bluer A_pu_ at 490 nm (see Figure S6 for its spectral intensity profile) and the higher resonance specificity of the 620 nm R_pu_ in conjunction with a bluer Raman probe on the anti-Stokes side to target PS*/PS*’ species (see the calculated tautomer ratios in Figure 1c) manifests a larger frequency shift toward the PS*/PS*’ tautomer, as well as a clear bidirectional ESIPT progression (less convoluted than the 645 nm R_pu_ case) from PA*’ to PS’* on the ~500 fs timescale (50–74% weight) and from PS*’ to PA’* on the ~1 ps timescale (46–53% weight, Figure 5).

A detailed examination of the observed peak frequency dynamics yields some interesting insights, corroborated by representative FSRS spectral comparisons with the best-fit Gaussian peak profiles at characteristic time delay points (Figure S7). Due to the negligible energy difference between the two tautomers in the non-equilibrium excited state (Scheme 1), the actual measured frequency can be best described as an average of nearly equal contributions from PA* and PS* vibrations; additional contributions may arise from an intermediate state (the proton is shared in the semi-ketonic state) on similar timescales as we revealed using fs-TA spectroscopy and the –OH-bond-coordinate-scan quantum calculations [46]. Any shifts observed from this initial region (beyond 200 fs) therefore mark changes in the population ratio from the nearly equipopulous S_1_. The blueshifts on the ~280–550 fs timescale could thus track the more downhill PA*→PS* (and less so for PA*’→PS*’) tautomerization, observed most clearly with the more PS* and PS*’-targeted 620 nm R_pu_ (Figure 5). This interpretation is supported by the rapid swing-back of both modes, red-shifting to approximately the same center frequency for the ~1296 cm^−1^ mixed-tautomer mode (Figure 5a) but more distinct frequencies for the ~1330 cm^−1^ mode with more PA* character (Figure 5b) by ~5 ps. Ground-state calculations predict the latter Raman mode to lie in a space between two regions of signal, with the nearest peak on either side belonging to PA (Figure 4c,d). Although there is some overlap of individual data points, the ~8 cm^−1^ difference between the least-squares fit lines is conspicuous, and this gap would be larger if not for the absence of the final redshift component with 537 nm A_pu_ and 645 nm R_pu_ (Figure 5b). The overall blueshift of the observed peak frequency blueshift from 250 fs to 5 ps (denoted by horizontal dashed lines in Figure 5) indicates vibrational cooling from PA* to PA*’ as the system navigates the potential energy surface into the relaxed ESIPT regime before the radiative and nonradiative relaxation pathways toward the electronic ground state (S_0_) become dominant [60,107,108].

Beyond 5 ps, the typical solvation-facilitated rotational relaxation time constant (~20 ps) and the apparent fluorescence lifetime (hundreds of ps to ~1 ns) dominate; more variance was observed due to weaker signal at long time delay points as well as the multiple relaxation pathways of the chromophore. These components mainly track further vibrational cooling processes as reflected by the Raman peak frequency blueshift in Figure 5. A noticeable redshift time constant of ~2.1 ps in the early part of this time regime for the 537 nm A_pu_ and 645 nm R_pu_ case may involve additional PA*’ formation via the slightly more uphill ESIPT transition pathway by transferring the proton closer to the methoxy sidechains since the associated activation energy is only ~33 meV higher than TS_1_ depicted in Scheme 1 (see Section 1) [46]. We note that the 490 nm A_pu_ paired with 620 nm R_pu_ case exhibits higher sensitivity to rotational relaxation, as the ~20 ps blueshift components have more pronounced weights of 7–14%, whereas the corresponding process is insignificant for both modes in the 537 nm A_pu_ paired with 645 nm R_pu_ case (Figure 5). This result generally correlates this process to excess photoexcitation energy with blue (vs. red) pump pulses to facilitate the more global chromophore-motion-induced vibrational cooling to reach the fluorescent state, in accord with the general trend for more global atomic displacements for the calculated PA-specific vibrational normal modes (see Figure S8) [61,107,108,109,110,111,112].

Furthermore, the redder peak frequency with 537 nm A_pu_ and 645 nm R_pu_ case substantiates the PA*/PA*’ species exhibiting an overall redder peak frequency than PS*/PS*’ species for both modes analyzed in Figure 5. In particular, both the starting and ending peak frequencies are similar for the mixed species mode (~1300 or 1298 cm^−1^ for both green and blue data points around the photoexcitation time zero or ~1 ns in Figure 5a), while there is a notable difference when a mostly PA* mode is tracked (~1334 or 1329 cm^−1^ for green and 1343 or 1338 cm^−1^ for blue data points around time zero or ~1 ns in Figure 5b), consistent with the tautomer state assignment. We suspect that if an alternative wavelength condition for all the incident pulses was found to achieve more targeted resonance enhancement with the PA*/PA*’ species only (instead of a mixture of both tautomers), the “doubly parabolic” peak frequency dynamics after 537 nm A_pu_ and 645 nm R_pu_ would give way to a single curve with a larger shift magnitude, mirroring the prominent parabola observed under the 490 nm A_pu_ and 620 nm R_pu_ condition. Nevertheless, the retrieved distinct excited-state Raman peak frequency shift patterns from ES-FSRS of two marker bands under two excitation conditions substantiates (1) the initial unrelaxed ESIPT reaction mainly for PS*→PA* (a bit more uphill than the PA*→PS* transition that likely occurs within the cross-correlation time of the optical setup) before ~250 fs, and (2) the rapidly relaxed ESIPT reaction that involves the PA*’→PS*’ transition on the 280–550 fs timescale and a slightly slower PS*’→PA*’ transition (more uphill than the PA*’→PS*’ transition, see Scheme 1) on the ~1 ps timescale of Draconin Red in solution. These characteristic excited-state Raman peak frequency shifts are also visible in global analysis of the ES-FSRS data via the correlated EADS and DADS (see acronyms in Figure 3 caption above) comparisons of vibrational signatures (Figure S4) [65,113]. Notably, this level of insights and distinction between PA*’→PS*’ and PS*’→PA*’ transitions are challenging to be directly obtained from fs-TA spectroscopy (see Section 2.1) due to the spectral overlap between broad electronic features and a net effect of ultrafast ESIPT events interconverting two tautomers; therefore, we mainly observe an average relaxed ESIPT time constant of ~600 fs for the more uphill PS*’→PA*’ pathway due to the bottleneck effect [46].

Additional insights can be obtained from a comparative analysis of four Raman marker band intensities under two excitation conditions. First, the general observation of the peak intensity decay and rise dynamics leads to a pronounced “W” pattern within the detection time window of ~1 ns, and the only exception is a lack of initial decay on the ~240–620 fs timescale for the 1296 cm^−1^ mode with 490 nm A_pu_ and 620 nm R_pu_ (Figure 6a). This result can essentially serve as a benchmark for the PA*’ formation on the 1.6 ps timescale with all the signal averaging effect from both tautomers in the relaxed ESIPT regime, consistent with the aforementioned ~1 ps timescale (particularly matching the 1296 cm^−1^ mode frequency redshift time constant of 1.6 ps toward the 10 ps time delay mark, see Figure 5a) and the bluer-light-induced overall larger PA*’ and PS*’ populations. Second, the largely conserved 1–2 ps rise component after the initial sub-ps decay (Figure 6b–d) is more pronounced (higher weight, green labels) with 537 nm A_pu_ and 645 nm R_pu_ than that with 490 nm A_pu_ and 620 nm R_pu_ (blue labels), except for the mixed mode at 1296 cm^−1^ (Figure 6a), corroborating the PS*’→PA*’ transition. Third, a longer intensity rise time constant of 6–10 ps is only present with 537 nm A_pu_ and 645 nm R_pu_ which can be rationalized by the pertinent SE peak redshift on a similar timescale (Figure 3b) that achieves more dynamic resonance enhancement [60,61,105]. Though the interplay between the tautomer population shift from electronic ground to excited state and resonance conditions adds complexity to a precise description of the observed Raman band intensity dynamics, some detailed conformational pathways can be summarized below.

The 1296 cm^−1^ mode, being in a frequency range that is calculated to be a mixture of PA and PS Raman modes, allows the PS-targeted 490 nm A_pu_/620 nm R_pu_ and the PA-targeted 537 nm A_pu_/645 nm R_pu_ conditions to illustrate the differences between tautomer relaxation behaviors. In the PA-targeted experiment, the initial 620 fs decay (82% weight) is followed by a prominent 900 fs rise (56% weight). In contrast, the PS-targeted experiment shows a prominent 1.6 ps rise (51% weight), followed by one 340 ps decay component that likely tracks the averaged long-time decay pathways from both PA*’ and PS*’ states (Scheme 1). The more-PA* 1330 cm^−1^ mode exhibits a similar relaxation fingerprint after both excitation conditions: a prominent sub-ps decay is followed by a ~1 ps rise (tracking a more uphill PS*’→PA*’ transition), and then a biphasic decay via rotational relaxation and other energy dissipation pathways. Moreover, the PA-targeted 537 nm A_pu_/645 nm R_pu_ condition enables an extra intensity rise (~180 fs, 6% weight) and a larger, slightly longer decay time constant (620 fs, 79% weight) to be retrieved, evident of a larger population of vibronically coupled PA*’ mode undergoing the relaxed and more downhill ESIPT for PA*’→PS*’, before the ~1.1 ps rise component (50% weight) that tracks the more uphill ESIPT for PS*’→PA*’ (Figure 6b). Raman modes in the 1400–1500 cm^−1^ ring deformation and COH-rocking regions (see Figure S8 for normal mode illustrations) also contain valuable information due to the influence of ketone group placement on the benzene ring motions. The 1430 cm^−1^ mode is predicted to have more PA*/PA*’ contribution while the 1488 cm^−1^ mode consists of more PS*/PS*’ contributions (Figure 4c,d and Figure S5). While these intensity fit lines make a broad “W” shape, the intensity and skewness of each U-curve demonstrates the nuanced behavior of both tautomer populations that comprise this unique Draconin Red chromophore [38,41,42,46]. For instance, the much more pronounced rise component on the 1.5–1.6 ps timescale for the 1296 and 1430 cm^−1^ modes after 490 nm A_pu_/620 nm R_pu_ excitation (see blue labels in Figure 6a,c) than other modes can be attributed to a significant dynamic resonance enhancement for PA*/PA*’ on the ps timescale: the corresponding Raman probe at 570–575 nm falls in the region for the initial SE peak redshift as highlighted by thin red arrows in Figure 2a as well as the gradient arrows in Figure 3a, thereby achieving notably enhanced Raman peak intensities [61,105].

For comparison, the ~1488 cm^−1^ mode has the PS* signal more easily separable from PA*, leading to additional cartographic insights into the excited-state potential energy surface (Scheme 1). While both tautomers likely still contribute to the Raman band in this region, a bluer peak is obtained with the 645 nm R_pu_ (Figure 4b), attributable to a calculated ground-state PS peak around 1470 cm^−1^ (Figure 4d), while the pertinent normal mode consists of more localized atomic motions on one side of the chromophore with both hydroxyl groups (Figure S8). After 537 nm A_pu_, a biphasic decay with 120 and 900 fs time constants is followed by a biphasic rise with 2.2 and 10 ps time constants (Figure 6d). After 490 nm A_pu_, an initial 340 fs decay is followed by a sharp 1.4 ps rise of equal magnitude (19% weight) to its 537 nm A_pu_ counterpart, implying that both PS*’ and PA*’ species contribute to this region (e.g., PS*’→PA*’ transition on the ~1 ps timescale). We note that the first decay component observed for this mode with 645 nm R_pu_ correlates to a state with a 120 fs lifetime, a time constant that has thus far only been seen as a rise in PA*-attributed probe window in the TA spectra (Figure 3c). Therefore, this time component likely tracks a slightly more uphill PS*→PA* transition (following a more downhill PA*→PS* transition masked by the laser pulse cross-correlation time, or direct excitation of PS species but to a lesser extent than 490 nm A_pu_), rationalizing this initial decay seen in the more PS*-attributed vibrational probe window. For comparison, the aforementioned 340 fs decay with a larger weight (77%) could arise from a rapid population shift from the more effectively pumped PS* species undergoing both PS*→PA* (120 fs) and PS*→PS*’→PA* (~1 ps) transitions, coupled with the electric polarizability change associated with the rapid molecular conformational change on the sub-ps timescale [47,114].

## 3. Perspectives and Conclusions

Since Draconin Red as a tree fungal pigment (from the spalting wood fungus *Scytalidium cuboideum*) is comprised of two fused aromatic rings with adjacent/opposing two carbonyls and two hydroxyl groups [38,46], our previously investigated blue-green pigment xylindein (from the spalting wood fungus *Chlorociboria aeruginosa*) provides an interesting and informative comparison [14,15,16,35]. The major photochemical relaxation pathway of xylindein comprises an ultrafast ESIPT (<200 fs, with a highly efficient unidirectional ESIPT of two away-from-tail hydroxyl groups toward their adjacent central carbonyl groups) and a rapid conformational “butterfly” twist of the rather flexible eight-fused-ring framework (with four carbonyls and two hydroxyl groups) on the ~30 ps timescale, resulting in an efficient and sloped S_1_/S_0_ conical intersection [16]. The larger molecular size and electronic π-conjugation of xylindein inherently leads to a smaller S_0_-S_1_ energy gap, increasing the probability for a nonradiative transition. In contrast, the compact Draconin Red chromophore essentially inhibits the conformational twisting pathway, and instead becomes more trapped in the electronic excited state beyond the initial “unrelaxed” ESIPT reaction (i.e., between PS* and PA*) by rapidly undergoing vibrational cooling toward a lower-lying electronic excited state S_1_′ (i.e., PS*’ and PA*’), enabling the observation of a “relaxed” bidirectional ESIPT reaction (i.e., PS*’↔PA*’) on the few ps timescale (likely facilitated by solvation dynamics of DCM solvent) before rotational relaxation on the ~25 ps timescale and other longer radiative and nonradiative pathways (see Scheme 1).

As further support, we measured the fluorescence quantum yield (FQY) of Draconin Red in DCM to be ~3.5% upon 500 or 530 nm excitation, which is more than one order of magnitude higher than the FQY of xylindein in DCM (<0.1% upon 532 nm excitation) [15,46]. This finding nicely correlates the more pronounced bidirectional ESIPT reaction pathways to the fluorescence pathway as both the accumulated and stabilized (vibrationally cooled) PA*’ and PS*’ species can emit, resulting in the clear vibronic structure of steady-state emission band from both tautomers with a ~605 nm peak (Figure 1c). Given the methoxy-sidechain-induced preference of TS_1_ (moving the distal proton as depicted in Scheme 1) and pertinent COH rocking/ring deformational motions across the high-frequency region (Figure S8), the substitution with a bulkier tail sidechain could lengthen the excited-state lifetime and hinder intermolecular interactions between Draconin Red molecules in condensed phase (e.g., solution and thin films), which may benefit its potential optoelectronic applications [2,16,46] and require further investigations from higher concentrations in solution to crystalline states. Another strategy entails the site-specific substitution to reduce the symmetry of Draconin Red, favoring one dominant tautomer in the electronic ground state with a prominent directional charge transfer if opposite sides of the chromophore are substituted with the electron-donating and -withdrawing groups, respectively [115,116,117].

Another relevant comparison can be made between the ESIPT observed in Draconin Red and a similar process in two well-known ESIPT dyes, indigo and alizarin [19,45]. In alizarin, the ESIPT must be unidirectional as there is only one prominent proton donor site (–OH) with an adjacent proton acceptor (C=O). Moreover, there is only one conformer present in the ground state, termed the 9,10-keto or locally excited (LE) state, because the proton transfer barrier is too high in the ground state. In the excited state proceeding the proton transfer, the 1,10-keto state forms which is referred to as the proton transferred (PT) state [19]. The ES-FSRS data yield a correlated frequency blueshift of the C=C stretch and redshift of the C=O stretch within ~150 fs of photoexcitation (on the 70–80 fs timescale) which was attributed to the LE→PT transition, especially due to the observed redshift that must be due to a structural change (ESIPT, and the altered resonance structure) instead of vibrational cooling (blueshift) [19]. Similar to alizarin, the monomeric form of indigo exists in two distinct conformers in the ground and excited states, the keto and enol conformers, respectively. However, in difference from alizarin, two ESIPT processes may occur in the indigo monomer due to the presence of two identical proton donor (–NH) and acceptor (C=O) moieties in the symmetrical molecule. While two ESIPT processes may occur, the ESIPT remains unidirectional because only one conformer (keto) exists in the ground state [45]. The complexity of analyzing and interpreting the ultrafast dynamics and ESIPT in Draconin Red is thus threefold as follows. First, Draconin Red exists in two conformers in the ground state, PA and PS, demonstrating different symmetries. Second, the ESIPT reaction can be bidirectional in that both PA* and PS* can undergo proton transfer in the excited state. Third, the tautomers can interconvert between each other via ESIPT by transferring one proton at a time but not two protons simultaneously (i.e., returning to itself) [46]. In other words, an ESIPT event starting from PS* can form PA* and vice versa. Another compounding factor adding complexity to interpreting the ultrafast dynamics of Draconin Red is that there are two similar but distinct ESIPT sites closer to and farther away from the two methoxy sidechains (see Figure 1a,b), which can be differentiated by TD-DFT calculations in the relaxed excited state predicting a slight energetic preference for transferring the proton farther away from the methoxy groups (see TS_1_ in Scheme 1) [46]. As a result, the unidirectional ESIPT process occurs between well-defined reactant and product conformers in alizarin and indigo, which is juxtaposed by the bidirectional ESIPT amongst a heterogeneous distribution of two ground-state conformers in Draconin Red, thereby posing considerable challenges for experimentation and spectral data interpretations in this work.

Using a combination of ultrafast electronic and vibrational spectroscopic techniques in tandem with quantum calculations, we have successfully extracted the tautomer-targeted dynamics of Draconin Red pigment in solution. Through the wavelength-tunable time-resolved FSRS technique, separable dynamics of the non-equilibrium excited-state potential energy surface were found that cannot be individually resolved from fs-TA experiments. Notably, the presence of two energetically distinct ESIPT-ready sites, an inherent heterogeneous chromophore population, and the bidirectionality of keto-enol ESIPT pathways for the tautomers to interconvert necessitates multiple resolvable ESIPT rates on the sub-ps to few ps timescales, aided by dynamic resonance enhancement insights afforded by the electronic dynamics of the two tautomers from fs-TA results. The use of a vibrationally resolved spectroscopic technique both enriches and refines the ESIPT pathways in Draconin Red, providing a bilateral survey of the routes the chromophore traverses in the excited state on intrinsic molecular timescales. While fs-TA spectroscopy typically resolves a single ESIPT-attributed decay component comprising a nominal average of downhill and uphill ESIPT below 1 ps, FSRS allows for the tracking of excited-state structural dynamics to resolve the bidirectional ESIPT reaction as a favorable 280–550 fs PA*’→PS*’ transition and a less favorable PS*’→PA*’ transition on the ~1 ps timescale, intimately related to the specific symmetry of each tautomer with respect to proton and ketone group placement. In the context of our prior TA-focused work [46] on Draconin Red, major advances and findings in this work can thus be summarized as follows: (1) Improved resolution of both the downhill and uphill ESIPT processes of Draconin Red in both the unrelaxed and relaxed temporal regimes prior to fluorescence. We found evidence that the uphill PS*’→PA*’ ESIPT time constant from fs-TA may be an average of the relaxed PA*’→PS*’ and PS*’→PA*’ tautomerization from ES-FSRS. (2) Capture of the excited-state Raman spectra of an ESIPT dye molecule during bidirectional proton transfer in action on ultrafast timescales. (3) Illustration of dynamic resonance enhancement using tunable FSRS technique to track a rapidly changing potential energy surface. (4) Achievement of preferential targeting of individual tautomers within a heterogeneous population of chromophores. The last point is rather unique since most ESIPT systems only have one dominant conformation in the ground state (see above). Furthermore, the strategic selection of the 485/490 nm actinic pump pulse used in this work has further enriched our proposed model on the photochemistry of Draconin Red [46] and provided evidence that a 510 nm laser pulse can excite both tautomer species to a large degree. With the bluer actinic pump, the ESIPT reaction can be tracked in a more unidirectional manner with deeper structural dynamics insights.

Such a powerful line of inquiry has profound implications for fundamental investigations of light-sensitive organic molecules in solution and condensed phases in general. Elucidation of this unique excited-state potential energy surface with prominent bidirectional ESIPT surviving vibrational cooling to a lower-lying electronic excited state demonstrates the power of a table-top spectroscopic toolset with wavelength tunability, which enables versatile targeting for specific components of a complex system. The gained structural dynamics insights into the structure–function relationships may act as a steppingstone toward the design and utilization of naturally sourced and sustainable organic chromophores for solar energy harvest and transfer in myriad device applications.

## 4. Materials and Methods

### 4.1. Sample Preparation

The Draconin Red samples used were prepared using the same methodology detailed in previous publications [38,118]. Currently, no organic synthesis route for Draconin Red has been previously established that could be found to the best of our knowledge [41,42]. Consequently, a culture of *Scytalidium cuboideum* (Sacc. & Ellis) Sigler & Kang (UAMH 11517) was matured on 2% malt agar (VWR, Radnor, PA, USA) plates with white-rotted maple wood chips for six weeks until fully pigmented, in accordance with the established protocols [12]. The media containing the fungal samples were air-dried and processed into a powder using an Oster blender (Model 6811). The HPLC-grade acetone at 99.9% purity (VWR) was then added to the powder to extract the pigment and the resulting mixture was stirred at room temperature (20 °C) before filtration through 415 filter paper (VWR). To increase pigment concentration, the solution was placed in a Büchi Rotavapor (Model 461) in a 40 °C deionized water bath to evaporate the acetone solvent. Crystallization of the pigment was then promoted by means of liquid nitrogen (following the established methods [38]), and filtered using the aforementioned 415 filter paper. The resulting crystals as previously shown to have high purity at 99.9% [118] were dissolved in acetone, while the remaining suspended crystal clumps were extracted using tweezers. Prior to all the experiments performed, dichloromethane (99% purity, TCI America, Portland, OR, USA) was added to vials of the dry crystal of Draconin Red to reach the desired optical density. The vials were lightly agitated to stimulate dissolution of the solid crystals before spectroscopic measurements.

### 4.2. Steady-State Electronic Spectroscopy

After dissolution into dichloromethane, the red pigment was transferred via micropipette into a 1-mm-pathlength quartz cuvette (Spectrosil 1-Q-1, Starna Cells, Inc., Atascadero, CA, USA) or a four-sided rectangular 5-mm-pathlength quartz cuvette for steady-state electronic absorption and emission measurements, respectively, at room temperature (~22 °C). Absorption spectra were collected with a Thermo Scientific Evolution 201 UV/Visible (UV/Vis) spectrophotometer (Thermo Fisher Scientific, Inc., Waltham, MA, USA), while emission spectra were acquired by a Shimadzu RF-6000 spectrofluorophotometer (Shimadzu Scientific Instruments, Pleasanton, CA, USA).

### 4.3. Femtosecond Transient Absorption (fs-TA) and Femtosecond Stimulated Raman Spectroscopy (FSRS) from the Ground to Excited State

For a thorough description of our tabletop home-built, ultrafast optical setup including both fs-TA and FSRS techniques, please refer to our prior publications (also containing the tunable pulses’ spectra, temporal profiles, and stabilities) [47,57,58,60,62,106]. A succinct description starts with a mode-locked Ti:Sapphire oscillator (Mantis-5) that seeds a regenerative laser amplifier (Legend Elite-USP-1K-HE, Coherent, Inc., Santa Clara, CA, USA). The fundamental laser output is a 1 kHz pulse train centered at ~800 nm with 35 fs pulse duration and 3.7 mJ pulse energy. The fs actinic pump for fs-TA and ES-FSRS is generated from a two-stage noncollinear optical parametric amplifier (NOPA) across a broadly tunable range (~480–720 nm) with sufficient power. To correct for dispersion and compress the actinic pump pulse duration to <100 fs, the laser beam is directed through a chirped mirror pair (DCM-12, 400–700 nm, Laser Quantum, Inc., part of Novanta Photonics, Stockport, UK); the representative spectral profiles for the actinic pump pulses at 490 and 537 nm (with characteristic FWHM values of ~340 and 570 cm^−1^, respectively) are shown in Figure S6. A synchronized chopper in the actinic pump beam path operates at half of the repetition rate of the fundamental (i.e., 500 Hz) to generate the difference signal in real time for data averaging and output via a custom-made LabVIEW suite. The probe beam for fs-TA and FSRS measurements is supercontinuum white light generated by focusing a portion of the fundamental laser onto a water-filled cuvette. The broadband probe is dispersion-corrected and compressed by another chirped mirror pair (DCM-9, 450–950 nm, Laser Quantum, Inc.). The temporally compressed actinic pump and probe pulses result in an average cross-correlation time of ~140 fs for both fs-TA and ES-FSRS experiments of Draconin Red in solution [46,62,74] as investigated in our optical setup; we note that the wavelength-dependent experimental response function (if applicable due to sample dispersion in the visible region) does not seem to exert any significant effect on our systematic spectral fitting and comparative data analysis between the electronic and vibrational dynamics under two contrasting excitation conditions (i.e., targeting PS and PA tautomers of Draconin Red) in this work (e.g., see Figure 2c–f, Figure 5 and Figure 6). Global analysis of fs-TA spectra was performed using the open-source software Glotaran [93].

Particularly for GS- and ES-FSRS, the ps narrowband Raman pump (R_pu_ as defined above) is produced by a three-stage NOPA system. First, an fs-NOPA generates the seed pulse at the desired wavelength, which is directed to a slit-based spectral filter to become a ps seed. Subsequently, a two-stage ps-NOPA is required to amplify the R_pu_ at 620 and 645 nm (using a ps 400 nm pump generated separately) to a high enough power and achieve a satisfactory signal-to-noise ratio of the stimulated Raman peaks [58]. A chopper is placed in the R_pu_ beam path, also synchronized at half of the main laser repetition rate (i.e., 500 Hz) according to the signal generation scheme, while a shutter is placed in the actinic pump beam path. All the incident laser pulses on the sample are *p*-polarized (i.e., the electric field is polarized parallel to the optical table). For GS-FSRS measurements, the actinic pump is completely blocked.

To acquire the spectral data, the pump and probe beams are spatially and temporally overlapped onto the sample housed in a 1-mm-pathlength quartz cuvette. The beams are focused onto the cuvette with a spot size of 0.15–0.2 mm diameter at the focal point using an off-axis parabolic mirror [62,112], and the temporal position of the Raman probe with respect to the Raman pump maximum is experimentally optimized using a solvent standard (e.g., cyclohexane) to achieve the best FSRS peak lineshape (without dispersive profiles or ringing) and highest mode intensities across the detection spectral window [47,73,119,120]. After passing through the cuvette, the pump beams are blocked while the transmitted probe is collimated and focused into a spectrograph (IsoPlane SCT-320, Princeton Instruments, Inc., part of Teledyne Princeton Instruments, Trenton, NJ, USA) that disperses the signal with a reflective grating before being imaged onto a CCD array camera (PIXIS:100F, Princeton Instruments, Inc.). For fs-TA measurements with a wider spectral window, a 300 grooves/mm grating was used with a 300 nm blaze wavelength. For FSRS measurements that require higher spectral resolution, a 1200 grooves/mm grating was used with a 300 nm blaze wavelength. The actinic pump power used in fs-TA experiments was ~0.25 mW for the 485 and 537 nm excitations. For ES-FSRS, the actinic pump power at 490 and 537 nm was ~0.3 and 0.25 mW, respectively, while the Raman pump power at 620 and 645 nm was ~4.2 and 3.0 mW. The sample solution was constantly stirred using a home-made miniature magnetic stir bar to ensure that fresh sample was irradiated throughout this experiment and to prevent sample degradation. The UV/Vis spectra were recorded and compared before/after the ultrafast laser experiments to confirm sample stability. For fs-TA and FSRS measurements, the sample concentrations were adjusted to make the Draconin Red pigment’s absorption peak optical density (OD) of ~1.4 per mm at 510 nm.

### 4.4. Quantum Calculations

Procedures used to calculate the vibronic spectra for assessment of steady-state absorption and emission spectra (see Figure 1c) can be found in our previous publication on Draconin Red [46]. To model the experimental GS-FSRS data, ground-state DFT geometric optimization of each tautomer was performed in Gaussian 16 using the CAM-B3LYP level of theory and 6-311+G(3df,2p) basis sets with implicit solvation (adopting the default polarizable continuum model for dichloromethane) [85]. The checkpoint files generated during structure optimization were then referenced to perform a Raman mode vibrational analysis for each tautomer of Draconin Red (Figure 1a,b). The frequencies of the resulting Raman spectra were first scaled by a literature value (0.967) for general comparison, this value was later replaced by a scaling factor of 0.964 to better match the observed Raman peak frequencies (Figure 4c,d) [121,122]. Representative Raman modes relevant to the aforementioned discussions are illustrated in Figure S8.

To generate the pre-resonance ground-state Raman spectra, we used the Franck–Condon method in Gaussian 16 software. Input frequencies to simulate experimental Raman pump conditions relative to each tautomer’s calculated resonance frequency were found according to the Gaussian recommendation, the results (being within a 1% difference in frequency) were averaged to use the same input frequencies for both calculations. Using the checkpoint files obtained from vibrational analysis of the optimized S_0_ and S_1_ electronic states of each tautomer with the B3LYP functional and 6-311+G(3df,2p) basis set, the Franck–Condon method produced theoretical Raman spectra of each tautomer while interacting with the 620 and 645 nm Raman pumps used for FSRS measurements in this work (Figure S5).

## Data Availability

All data needed to evaluate the conclusions in the paper are present in the paper and the Supplementary Materials.

## Supplementary Materials

The following supporting information can be downloaded at: https://www.mdpi.com/article/10.3390/molecules28083506/s1, Figure S1: Comparison of selected transient absorption spectra to corresponding ES-FSRS spectra and GS-FSRS with manually drawn FSRS baselines of Draconin Red in DCM solvent; Figure S2: Intensity dynamics of selected vibrational modes (1296, 1330, 1430, and 1488 cm^−1^ in panels a–d) of Draconin Red using specific tautomer-targeted 485 nm actinic pump and 645 nm Raman pump; Figure S3: Intensity dynamics of the 1600 cm^−1^ Raman mode of Draconin Red after 490 nm actinic/620 nm Raman pump and 537 nm actinic/645 nm Raman pump; Figure S4: Global analysis of the ES-FSRS spectra of Draconin Red in DCM solvent under two representative excitation conditions; Figure S5: Pre-resonance experimental and calculated ground-state Raman spectral comparisons for two tautomers of Draconin Red in DCM solvent; Figure S6: Actinic pump spectral profiles in TA and FSRS experiments; Figure S7: Representative peak shifts of Raman marker bands in ES-FSRS spectra; Figure S8: Calculated Raman modes with atomic displacements for both tautomers of Draconin Red in DCM solvent; and Supplementary References. References [46,59,60,61,65,105,106,121,123,124,125,126] are cited in the supplementary materials.
