# Supplementary material for: Photogrammetry of Ultrafast Excited-State Intramolecular Proton Transfer Pathways in the Fungal Pigment Draconin Red
Source: Molecules. 2023 Apr 16;28(8):3506. doi: 10.3390/molecules28083506 (PMC10144053; doi:10.3390/molecules28083506)
Supplement: Supplementary file 1 [file molecules-28-03506-s001.zip › molecules-2309795-supplementary.pdf]

Article

Photogrammetry of Ultrafast Excited-State Intramolecular Proton Transfer Pathways in the Fungal Pigment Draconin Red

Janak Solaris, Taylor D. Krueger, Cheng Chen and Chong Fang *,†

Department of Chemistry, Oregon State University, 153 Gilbert Hall, Corvallis, OR 97331, USA; dunnwalj@oregonstate.edu (J.S.); kruegeta@oregonstate.edu (T.D.K.); chenc9@oregonstate.edu (C.C.)

* Correspondence: chong.fang@oregonstate.edu; Tel.: +1-541-737-6704

† Web: https://fanglab.oregonstate.edu/ (accessed on 13 April 2023).

Table of Contents

1. Supplementary Figures

Figure S1. Comparison of selected transient absorption spectra to corresponding ES-FSRS spectra and GS-FSRS with manually drawn baselines of Draconin Red in DCM solvent.. S2

Figure S2. Intensity dynamics of selected vibrational modes of Draconin Red using specific tautomer-targeted 485 nm actinic pump and 645 nm Raman pump ……………………..…..…S4

Figure S3. Intensity dynamics of the 1600 cm^{-1} Raman mode of Draconin Red after 490 nm actinic/620 nm Raman pump and 537 nm actinic/645 nm Raman pump……………..S6

Figure S4. Global analysis of the ES-FSRS spectra of Draconin Red in DCM solvent under two representative excitation conditions …………………………...…………………….. S8

Figure S5. Pre-resonance experimental and calculated ground-state Raman spectral comparisons for two tautomers of Draconin Red in DCM solvent …..………………….. S10

Figure S6. Actinic pump profiles in TA and FSRS experiments ……………………..……. S12

Figure S7. Representative peak shifts of Raman marker bands in ES-FSRS spectra…..... S13

Figure S8. Calculated Raman modes with atomic displacements for both tautomers of Draconin Red in DCM solvent …………………………………….………………….... S14

2. Supplementary References ……………………………..……………...…..…S16

1. Supplementary Figures

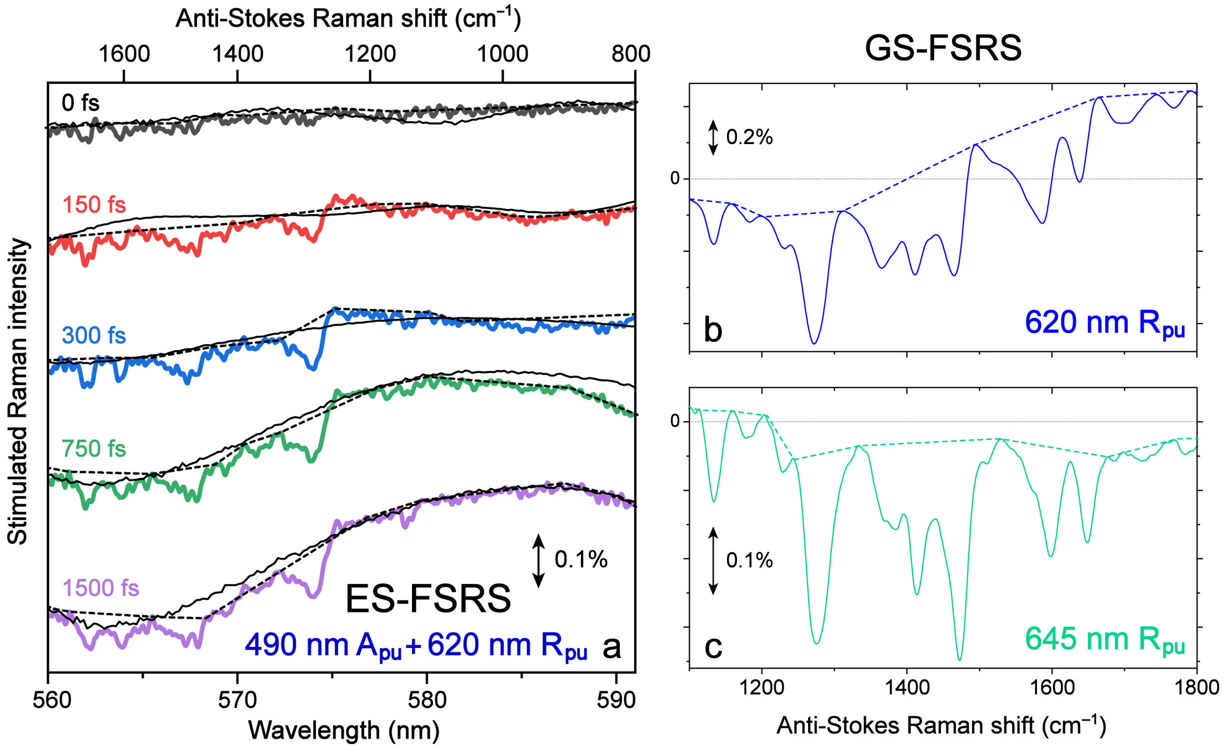

Figure S1. Selective excited-state (ES)- and ground-state (GS)-FSRS data of Draconin Red in DCM solvent. (**a**) ES-FSRS data after the solvent and ground-state correction before baseline subtraction, color-coded at five time delay points (0, 150, 300, 750, and 1500 fs), obtained using a 490 nm actinic pump (A_{pu}) and 620 nm Raman pump (R_{pu}). Solid black lines denote the femtosecond transient absorption (fs-TA) spectra measured using the same actinic pump and white-light probe at the corresponding time delay points (listed on the left side). Dashed black lines depict the consistently drawn baselines subtracted to obtain the excited-state Raman spectra mostly free of the TA background [1-3]. The probe wavelength in nanometer (nm) unit is shown in the bottom horizontal axis, while the corresponding anti-Stokes Raman shift in wavenumber (cm^{-1}) unit with respect to the redder Raman pump at 620 nm is shown in the top horizontal axis. The fs-TA spectra are normalized for easy comparison with the corresponding ES-FSRS spectra. Representative GS-FSRS data collected using (**b**) 620 nm and (**c**) 645 nm Rpu after the solvent

background correction. The color-coded dashed lines indicate the spectral baselines subtracted to obtain the ground-state Raman spectra shown in Figure 4c,d (see main text). The zero line is shown by the gray dotted line, exposing the negative signal under the experimental condition. The double-headed vertical arrow represents a stimulated Raman intensity magnitude of 0.1% in panel **a**, as well as 0.2% and 0.1% in panels **b** and **c**, respectively. This comparison showcases much smaller excited-state Raman peak intensities than their ground-state counterparts.

Notably, the spectral baseline for the GS-FSRS spectrum with 620 nm R_{pu} in panel **b** is more curved since the bluer Raman probe overlaps more with the ground-state absorption band (see Figure 1c in main text) than the 645 nm R_{pu} case in panel **c**. This finding further substantiates the resolving power of FSRS as a structure-sensitive technique with broadly tunable laser pulses to delineate rich information on the multidimensional potential energy surface of functional molecular systems from the ground to excited states [1-3].

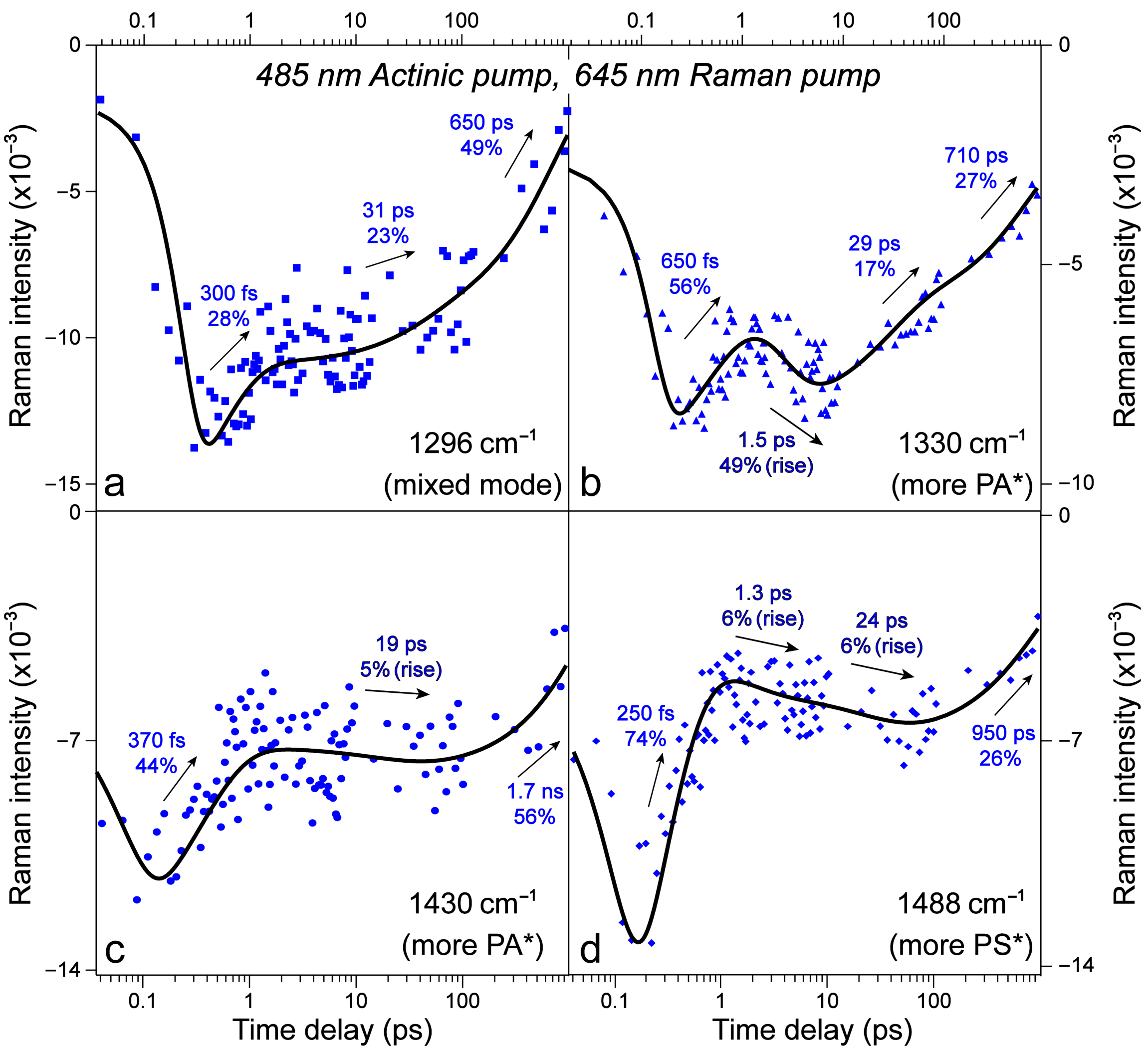

Figure S2. Excited-state Raman peak intensity dynamics of Draconin Red in DCM solvent following a PS-targeted 485 nm actinic pump and a PA-targeted 645 nm Raman pump. Dynamics reflect the integrated area of gaussian peak used to fit the transient Raman band at each time delay. Spectral data points (blue scattered symbols) are overlaid with the least-squares multi-exponential fits (solid black curves), with the retrieved decay and rise (explicitly denoted) components listed in the insets by their respective arrows. The amplitude weights for all the decay components sum to 100%, and the amplitude weight for each rise component is scaled accordingly.

Notably, the ~1296 and 1330 cm^{-1} Raman bands show the initial rise before ~200 fs due to the "unrelaxed" PS*→PA* more uphill transition (see Scheme 1), indicating that a significant

population of PS* can be generated by the 485 nm pump. In all four Raman modes in Figure S2, a 250–650 fs decay time constant is observed, in accord with the proposed rate of a more downhill ESIPT reaction from PA*' to PS*' ("relaxed" ESIPT) which is faster than the bottleneck of 400–750 fs reported previously (i.e., tracking an overall uphill ESIPT reaction from PS*' to PA*') [4]. The characteristic peak intensity rise beyond 1 ps that is responsible for the notable "W"-shaped dynamics is observed only in the 1330 and 1488 cm^{-1} modes, with the 1.5 ps (49% weight) and 1.3 ps (6% weight) rise components, respectively (see Figure S2b,d). This feature can be attributed to a more uphill ESIPT reaction from the relaxed PS*' to PA*' states than the more downhill and faster ESIPT reaction (i.e., PA*' to PS*'). The relative magnitudes of these rise components are consistent with the scenario that despite less specific targeting by the 645 nm Raman pump (Figure 1c), the small resonance favorability of PA*' over PS*' species impacts the observed dynamics as the overall excited-state population shifts to PA*' by a noticeable extent (more prominent for the 1330 cm^{-1} mode that has more PA* character, see Figure 4c,d). This trend can be viewed in the stimulated emission (SE) peak redshift on the sub-ps to ps timescale in fs-TA spectra (Figure 3a,b).

The rotational dynamics component shows up as a rise in the 1430 and 1488 cm^{-1} modes, but at a low magnitude (5–6%). The only other condition showing such a rise has 537 nm actinic pump with 645 nm Raman pump for the 1488 cm^{-1} mode (Figure 6d) but not the 1430 cm^{-1} mode (Figure 6c). This pattern may be related to a large data spread in the 1430 cm^{-1} case (Figure S2c), but it is generally reflective of a different mixing of various excited-state tautomeric species after 485 and 537 nm excitations (e.g., a redder actinic pump with a redder Raman pump manifests a 10–24 ps rise due to dynamic resonance enhancement [5]). Finally, the longest time constants exhibit no major pattern across the Raman bands, while their common range (0.7–1.7 ns) is in accord with a mixture of radiative and nonradiative pathways from the relaxed PA*' and PS*' species [4].

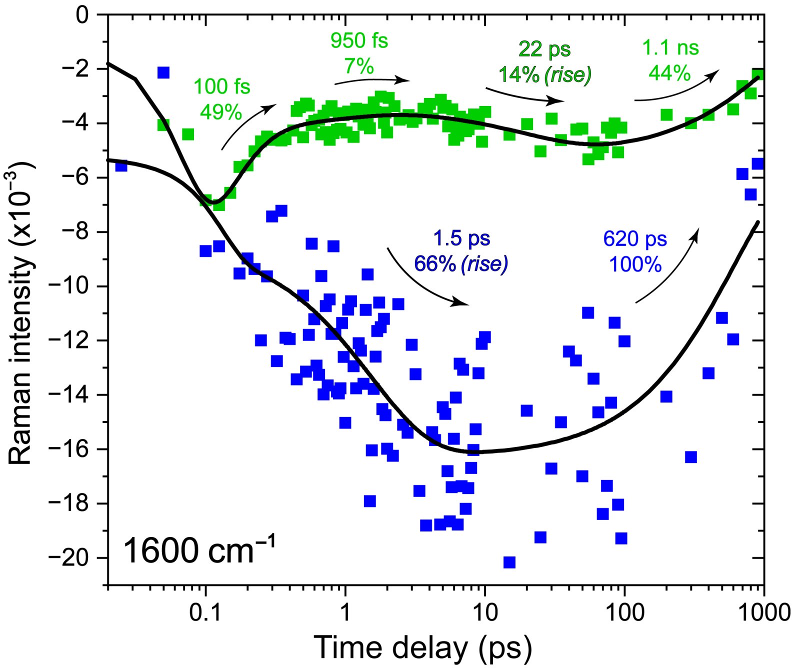

Figure S3. Intensity dynamics of the 1600 cm^{-1} Raman mode of Draconin Red in DCM solvent after 490 nm actinic pump with 620 nm Raman pump (blue) and 537 nm actinic pump with 645 nm Raman pump (green). Dynamics reflect the integrated area of gaussian peak used to fit the transient Raman band (showing up as a Raman loss signal due to the anti-Stokes experimental conditions used [3,5-7]) at each time delay. Spectral data points (blue and green scattered squares) are overlaid with the least-squares multi-exponential fits (solid black curves), with the retrieved decay and rise (explicitly denoted) components listed in the inset by their associated curved arrows. The amplitude weights for all the decay components sum to 100% under each excitation condition, and the amplitude weight for each rise component is scaled accordingly.

Due to the capricious nature of the TA background in this spectral region (i.e., 564 or 585 nm) with a 620 or 645 nm Raman pump following photoexcitation (e.g., see Figure S1), the spread of the weak signal taken is large, precluding robust analysis. The dynamics observed most resemble those seen in the mixed 1296 cm^{-1} mode (see Figure 6a in main text): the PS*'-targeted 620 nm Raman pump extricates two major components, while the PA*'-targeted 645 nm Raman pump

shows four components. Notably, the entire signal decay can be fit with an apparent fluorescence lifetime of 620 ps, preceded by a ~1.5 ps rise component (see blue data points and fit). In the redder pump case, the data spread is much smaller, increasing the reliability of components retrieved from the fit. Notably, the initial decay of the 1600 cm^{-1} mode (~100 fs time constant, 49% weight) is much faster than the 1296 cm^{-1} mode (~620 fs, 82% weight; mixed species), which indicates that the C=C/C=O stretching motion of PA* (more populated by the 537 nm actinic pump and resonantly enhanced by the 645 nm Raman pump) undergoes a more downhill transition to PS* in the unrelaxed ESIPT regime on the ultrafast timescale (<120 fs, see Scheme 1) [4].

Interestingly, two intermediate components (~900–950 fs, and 22–24 ps) observed in this mode after the 537/645 nm actinic/Raman pump show a sign flip relative to the 1296 cm^{-1} mode (Figure 6a), indicative of the mode- and tautomer-dependent vibrational dynamics pattern that arises from the intricate excited-state potential energy surface with vibronic structure [3,7]. Since the ~950 fs component likely occurs during relaxed ESIPT, tracking the more uphill PS*'→PA*' transition, the apparent signal rise to a much higher intensity before ~10 ps after 490 nm excitation (than the redder 537 nm excitation case) indicates that the 1600 cm^{-1} mode intensity mainly tracks the PA*' accumulation on the ps timescale. Moreover, the small yet noticeable ~22 ps rise component after 537 nm excitation is consistent with the dynamic enhancement of PA*' species [5] (see an SE peak redshift on the ~28 ps timescale around 565 nm in Figure 3b, achieving better resonance condition with the Raman probe that corresponds to the 1600 cm^{-1} mode) even though the overall excited tautomer populations are reduced vs. the bluer pump at 490 nm (Figure S3). Taken together, the weak Raman band at 1600 cm^{-1} can act as a structural probe for the bidirectional ESIPT reaction on ultrafast timescales, with bluer and redder excitations that can respectively highlight the relaxed and unrelaxed ESIPT reactions due to the swift tautomerization of Draconin Red in solution.

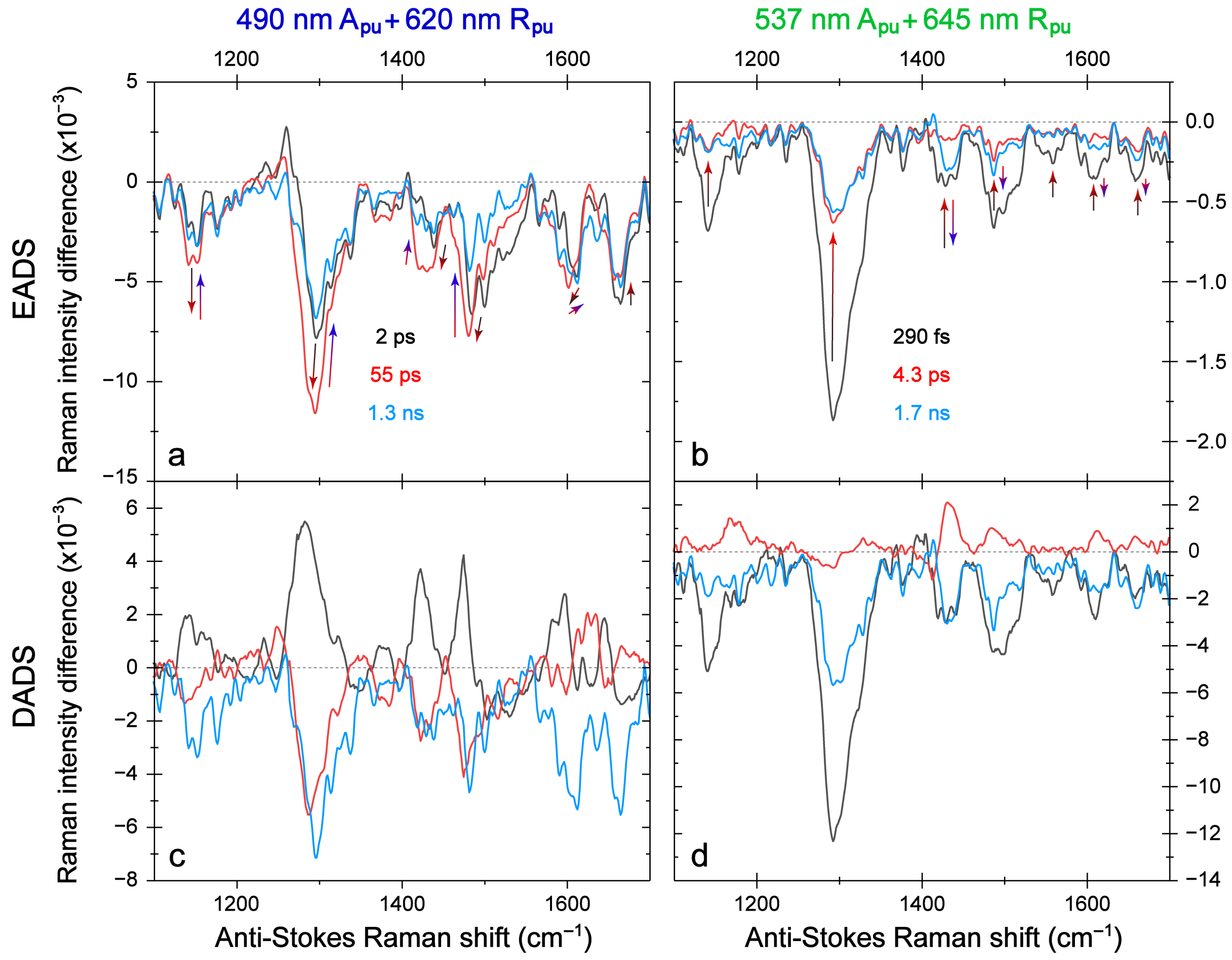

Figure S4. Global analysis of the ES-FSRS spectra on the anti-Stokes side of Draconin Red in DCM solvent obtained using a 490 nm actinic pump (A_{pu}) with a 620 nm Raman pump (R_{pu}, left panels) and a 537 nm A_{pu} with a 645 nm R_{pu} (right panels). The evolution-associated difference spectra (EADS) with (**a**) 620 nm and (**b**) 645 nm R_{pu} expose key changes in the excited-state Raman spectra after photoexcitation, color-coded (black→red→cyan) with the retrieved lifetimes listed in the insets. The gradient arrows depict the spectral peak evolution across the detection window. The decay-associated difference spectra (DADS) after (**c**) 490 nm and (**d**) 537 nm excitations provide further insights by utilizing a parallel model with identical lifetimes as EADS. Species being created cause an intensity magnitude increase in EADS and a positive signal in

DADS since the observed excited-state Raman peaks are negative under the experimental conditions used in ES-FSRS for Draconin Red in DCM solvent (see Section 4.3 in main text).

The spectra with a ~2 ps lifetime in Figure S4a,c indicate the creation of a species contributing to the Raman bands at ~1140, 1296–1330, 1430, 1490, and 1600 cm^{-1}. The initial redshift and subsequent blueshift of the EADS (see gradient arrows in Figure S4a), together with the discussion of Figure 5 in main text, imply a population shift from PS*’ to PA*’ (i.e., a more uphill transition) in the relaxed ESIPT regime around this time (Scheme 1). The intensity rise and redshift of the 1296 cm^{-1} marker band in the EADS (black→red traces in Figure S4a) affirm this process [4]. On the 55 ps timescale, the noticeable peak blueshift tracks vibrational cooling toward PS*’ species.

In contrast, following 537 nm excitation, a ~300 fs lifetime is observed, likely corresponding to a more downhill and favorable PA*’→PS*’ transition, followed by a 4.3 ps lifetime that involves more uphill PS*’→PA*’ conversion in conjunction with solvation dynamics [3,4]. The visual similarities between this 4.3 ps spectrum (red, Figure S4d) and the 2 ps spectrum after 490 nm excitation (black, Figure S4c) are striking. Once again, a species is being formed [8,9], leading to positive DADS peaks at ~1330, 1430, 1490, and 1610 cm^{-1}. The similarities between spectra in the first few ps following both excitations provide further evidence that the same ESIPT process occurs on this timescale: the relaxed uphill formation of PA*’ from PS*’. The differences between these two spectra, most notably the sign switch of 1296 cm^{-1} peak and shift of the positive feature from 1140 cm^{-1} (black) in the 490 nm excitation case to 1170 cm^{-1} (red) after 537 nm excitation indicates that the 2 ps spectrum following 490 nm excitation contains features that may be better resolved if an earlier spectrum could be generated for this experiment. This finding is corroborated by the negative DADS features across the detection window with the longest lifetime, including two broader peak doublets in the 1600–1660 cm^{-1} and 1100–1200 cm^{-1} ranges (Figure S4).

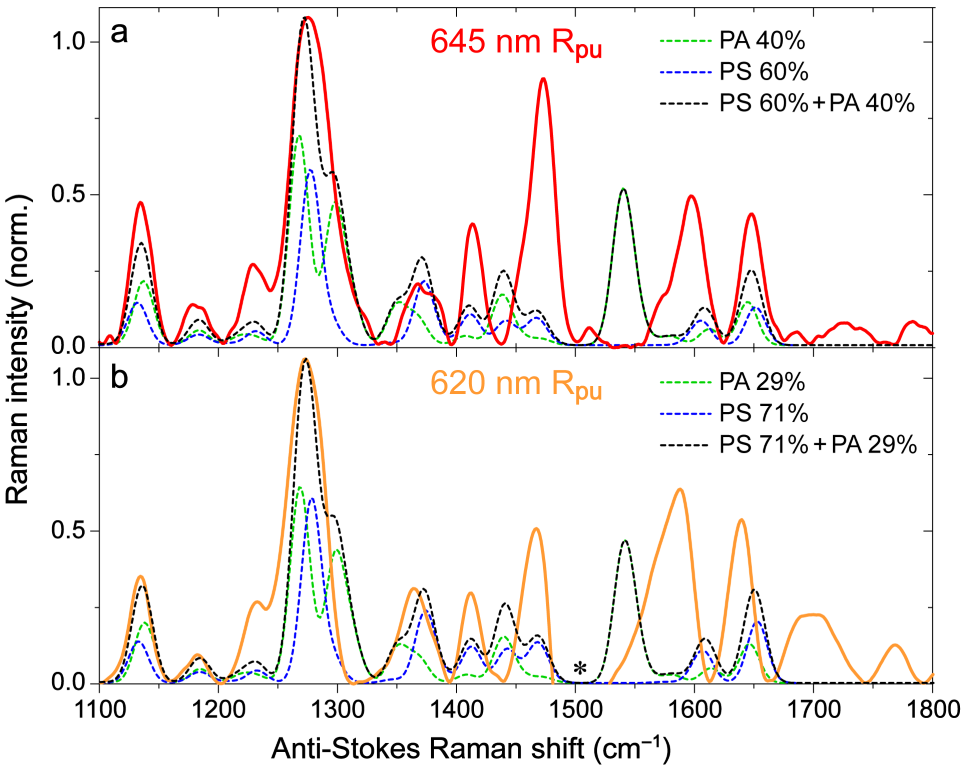

Figure S5. Pre-resonance experimental and calculated ground-state Raman spectral comparisons for two tautomers of Draconin Red in DCM solvent. Using the Franck-Condon method (see Section 4.4 in main text), the Raman spectra for PA and PS tautomers interacting with the 620 and 645 nm R_{pu} were calculated and scaled to compare with the experimental GS-FSRS data collected at the corresponding pre-resonance conditions (i.e., 620 and 645 nm that are redder to the main absorption peak at ~510 nm, see Figure 1c). The results were scaled according to the estimated ground-state tautomer populations (62% and 38% for PS and PA, respectively) and the estimated resonance preference at each R_{pu} location (i.e., 645 nm R_{pu}: 48% PS, 52% PA; 620 nm R_{pu}: 60% PS, 40% PA) and summed (black dashed line). The resultant spectra were frequency-scaled according to a literature value (0.967), which was then replaced by a scaling factor of 0.985 to better match the experimental spectra (thick red and orange lines in panels **a** and **b**). The baselines for the experimental FSRS spectra were redrawn (vs. the ones leading to GS-FSRS spectra in

Figure 4c,d) to highlight the Raman bands between ~1350–1500 cm^{-1} for better peak intensity comparison with the calculated pre-resonantly enhanced Raman modes. The asterisk in panel **b** denotes the region for a dispersive peak (the negative portion is not shown), since the experimental intensity is multiplied by –1 for comparison to the calculated spectrum with positive intensity.

The agreement between the calculated tautomer population- and resonance-weighted summation spectra (black) and the experimentally obtained pre-resonance Raman spectra (red and orange lines) is most evident in the ~1140, 1275, and 1380 cm^{-1} regions. The calculated PA peak intensity at ~1540 cm^{-1} (the only prominent peak between ~1500–1620 cm^{-1}) is similar in magnitude to the experimentally observed peak at ~1600 cm^{-1} with 645 nm R_{pu} (Figure S5a), suggesting certain limitations of our calculations to accurately predict all the mode frequencies. In particular, this discrepancy could arise from an overestimation of anharmonic effects, a solvation effect that is not adequately incorporated with a polarizable continuum model, or a vibronic coupling matrix that lacks accuracy [10-12]. Interestingly, the aforementioned excited-state peak intensity dynamics for the ~1600 cm^{-1} mode (see Figure S3 above) indicates the dominant contribution from PA*' species, in accord with a calculated prominent ground-state PA peak of Draconin Red in DCM solvent in this spectral region.

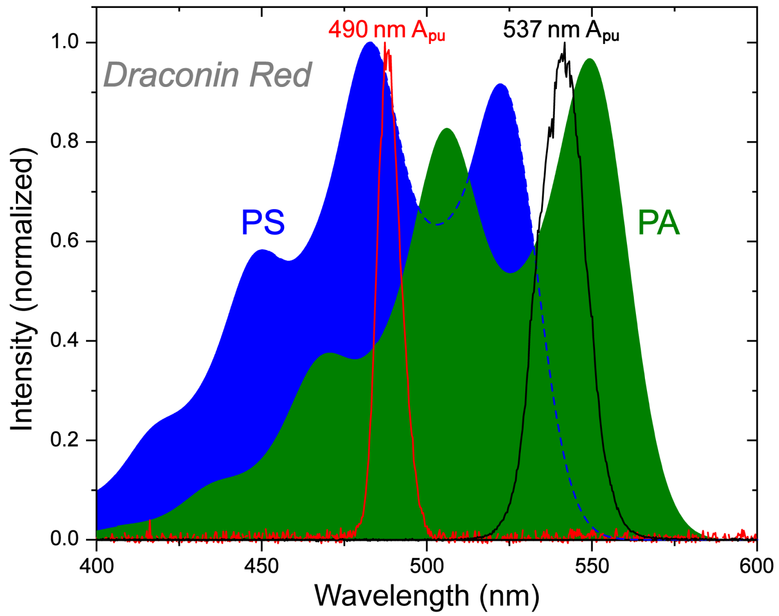

Figure S6. Representative intensity profiles of 490 nm (red) and 537 nm (black) actinic pumps (A_{pu}) plotted alongside the predicted electronic absorption spectra for PS (blue) and PA (green) tautomers of Draconin Red in solution. The A_{pu} profiles are normalized (showing largely gaussian profiles), while the absorption spectra are normalized to the maximum absorption band of both tautomers to preserve their relative intensities.

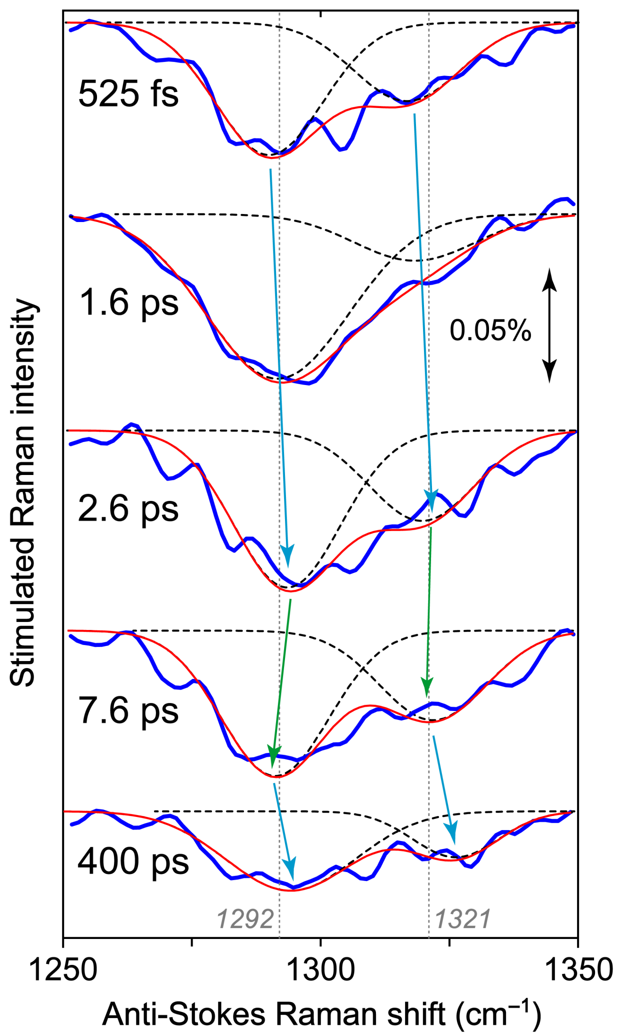

Figure S7. Representative peak shifts of Raman marker bands in ES-FSRS spectra. The baseline-subtracted excited-state Raman spectra (solid blue lines) of the Draconin Red pigment in DCM solvent are obtained using a 490 nm actinic pump and 620 nm Raman pump (see Figures 4a, 5 and 6 in main text). Dashed black lines denote the gaussian lineshapes used to fit two adjacent Raman marker bands within the 1250–1350 cm^{-1} spectral region at certain time delay points (listed). The summation of best-fit gaussian profiles is depicted in red. Dotted gray lines at both 1292 and 1321 cm^{-1} have been added to highlight center frequency shifts of these two Raman modes (marked by the tilted downward cyan and green arrows for the peak frequency blueshift and redshift, respectively; see the complete data plots across the detection time window in Figure 5, main text).

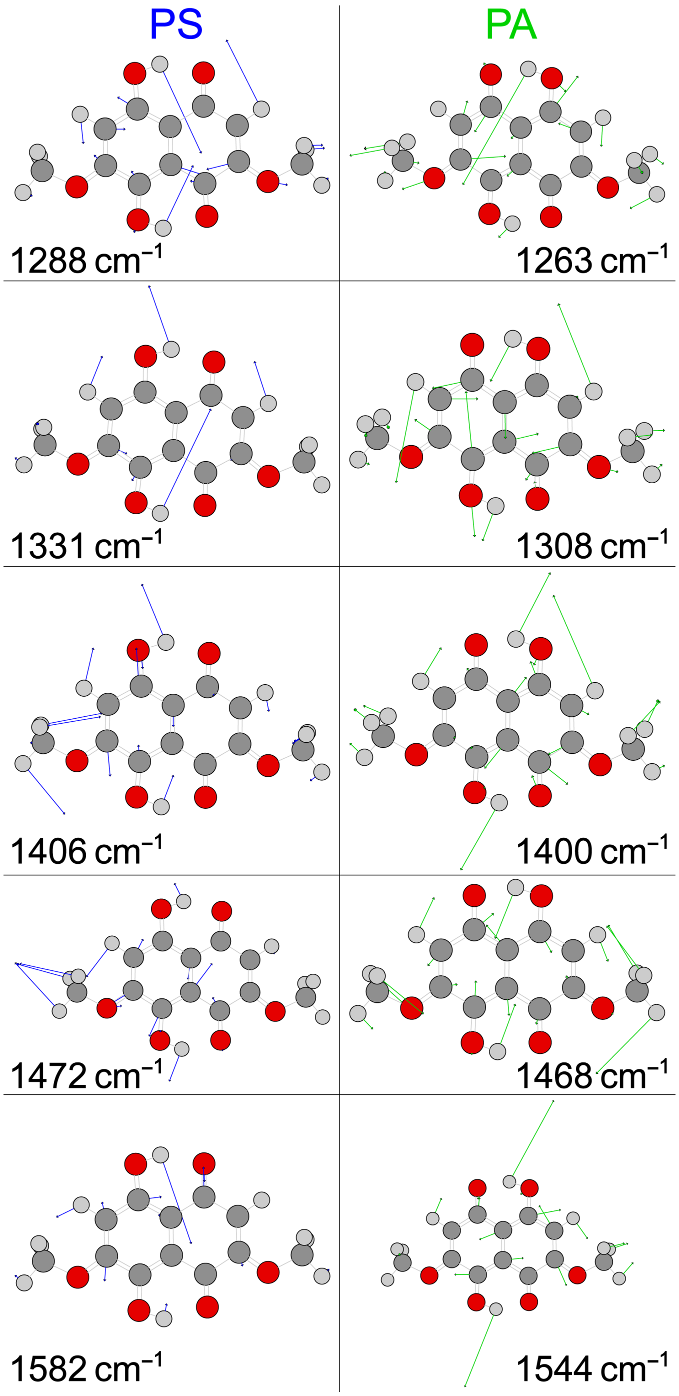

Figure S8. Calculated key Raman modes for the PS and PA tautomers of Draconin Red in DCM solvent using the CAM-B3LYP level of theory and 6-311G+(3df,2p) basis sets with an implicit solvation model. Vibrational frequencies were scaled by 0.964 to better match measured spectra. Each mode depicted is predicted to play a role in the excited-state FSRS spectra (see Figure 4a,b)

and the associated dynamics shown in Figures 5 and 6 (main text). Colored arrows depict the atomic displacements of each mode (blue: PS, in left panels; green: PA, in right panels), to scale with the relative intensity of other atomic displacements of the vibrational normal mode. With the exception of sidechain hydrogen displacements, all nuclear displacements of appreciable magnitude are within the plane of conjugation of the molecule. Atomic colors are as follows: C, dark gray; O, red; H, light gray.

2. Supplementary References

1. Dietze, D.R.; Mathies, R.A. Femtosecond stimulated Raman spectroscopy. *ChemPhysChem* **2016**, *17*, 1224–1251.
2. Liu, W.; Wang, Y.; Tang, L.; Oscar, B.G.; Zhu, L.; Fang, C. Panoramic portrait of primary molecular events preceding excited state proton transfer in water. *Chem. Sci.* **2016**, *7*, 5484-5494.
3. Fang, C.; Tang, L.; Chen, C. Unveiling coupled electronic and vibrational motions of chromophores in condensed phases. *J. Chem. Phys.* **2019**, *151*, 200901.
4. Krueger, T.D.; Solaris, J.; Tang, L.; Zhu, L.; Webber, C.; Van Court, R.C.; Robinson, S.C.; Ostroverkhova, O.; Fang, C. Illuminating excited-state intramolecular proton transfer of a fungi-derived red pigment for sustainable functional materials. *J. Phys. Chem. C* **2022**, *126*, 459-477.
5. Oscar, B.G.; Chen, C.; Liu, W.; Zhu, L.; Fang, C. Dynamic Raman line shapes on an evolving excited-state landscape: Insights from tunable femtosecond stimulated Raman spectroscopy. *J. Phys. Chem. A* **2017**, *121*, 5428-5441.
6. Liu, W.; Tang, L.; Oscar, B.G.; Wang, Y.; Chen, C.; Fang, C. Tracking ultrafast vibrational cooling during excited state proton transfer reaction with anti-Stokes and Stokes femtosecond stimulated Raman spectroscopy. *J. Phys. Chem. Lett.* **2017**, *8*, 997–1003.
7. Roy, K.; Kayal, S.; Ariese, F.; Beeby, A.; Umapathy, S. Mode specific excited state dynamics study of bis(phenylethynyl)benzene from ultrafast Raman loss spectroscopy. *J. Chem. Phys.* **2017**, *146*, 064303.
8. Berera, R.; van Grondelle, R.; Kennis, J.M. Ultrafast transient absorption spectroscopy: Principles and application to photosynthetic systems. *Photosynth. Res.* **2009**, *101*, 105-118.

9. Wang, Z.; Zhang, Y.; Chen, C.; Zhu, R.; Jiang, J.; Weng, T.-C.; Ji, Q.; Huang, Y.; Fang, C.; Liu, W. Mapping the complete photocycle that powers a large Stokes shift red fluorescent protein. *Angew. Chem. Int. Ed.* **2022**, *62*, e202212209.

10. Jensen, L.; Schatz, G.C. Resonance Raman scattering of rhodamine 6G as calculated using time-dependent density functional theory. *J. Phys. Chem. A* **2006**, *110*, 5973-5977.

11. Merrick, J.P.; Moran, D.; Radom, L. An evaluation of harmonic vibrational frequency scale factors. *J. Phys. Chem. A* **2007**, *111*, 11683-11700.

12. Quincy, T.J.; Barclay, M.S.; Caricato, M.; Elles, C.G. Probing dynamics in higher-lying electronic states with resonance-enhanced femtosecond stimulated Raman spectroscopy. *J. Phys. Chem. A* **2018**, *122*, 8308-8319.
